# Drivers of Soil Community Composition in Apple Orchards: Insights From Environmental Metabarcoding

**DOI:** 10.1111/1758-2229.70422

**Published:** 2026-10-05

**Authors:** Ulrike Obertegger, Emanuela Coller, Sara Zanoni, Roberto Zanzotti, Raffaella Morelli, Massimo Pindo, Loris Marchel, Daniele Prodorutti

**Affiliations:** ^1^ Fondazione Edmund Mach, Research and Innovation Centre Research Group Hydrobiology San Michele all'Adige Italy; ^2^ National Biodiversity Future Center—NBFC Palermo Italy; ^3^ Organic Agriculture and Soil Quality Unit Fondazione Edmund Mach, Technological Transfer Centre San Michele all'Adige Italy; ^4^ Fondazione Edmund Mach, Research and Innovation Centre, Computational Biology San Michele all'Adige Italy; ^5^ APOT, Associazione Produttori Ortofrutticoli Trentini Trento Italy

**Keywords:** community assembly, dbMEM, dispersal, Faprotax, FunGuild

## Abstract

Soil biodiversity in apple orchards plays a vital role for productivity. We studied the biodiversity of prokaryotes, protists and fungi of 26 apple orchards belonging to the same regional growers' association in Northern‐Italy by environmental DNA metabarcoding (16S, 18S, ITS). We assessed alpha‐ and beta‐diversity, distance decay relationships, immigration rates and community assembly processes, variance partitioning between space and environment and putative prokaryotic and fungal functions from the upper 20 cm of soil during summer 2023. Despite uniform management practices, orchards showed a huge variability in alpha diversity, with single orchards having only a small part of total diversity. Protists showed the lowest mean beta‐diversity, followed by prokaryotes and fungi. Although immigration rates were low across all organismal groups—reflecting the low number of taxa co‐occurring across all orchards—prokaryotes and protists still displayed relatively higher rates than fungi, in support of the size‐plasticity hypothesis. Community assembly was governed primarily by stochastic processes, even though deterministic processes were also important. Turnover dominated community composition for all organismal groups. Putative functions of prokaryotes pertained mainly to carbon and nitrogen cycling. Our study provided mechanistic insights into the diversity of soil organisms across different trophic levels found in apple orchards.

## Graphical abstract

Despite uniform management practices, single orchards of the 26 studied had only a small part of total diversity of prokaryotes, protists and fungi. Community assembly was governed primarily by stochastic processes and turnover dominated community composition for all organismal groups.

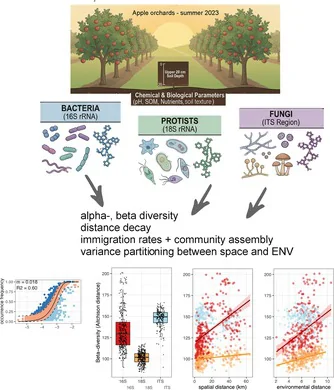

## 1 Introduction

Agricultural productivity and crop quality are fundamentally shaped by the soil's physical, chemical and biological properties (Bardgett and van der Putten 2014; Delgado‐Baquerizo et al. 2020; Xing et al. 2025). Microbial diversity is an important component of soil (Lehmann et al. 2020) as microbes provide essential ecosystem services such as organic matter decomposition, humification and biogeochemical nutrient cycling. Furthermore, microbes can promote mutualistic relationships with associated plants (Oldroyd 2013), inhibit or alleviate plant diseases (Liu et al. 2020) or act as plant pathogens (Peng et al. 2021).

The interconnections of soil biodiversity, plants and the environment determine complex outcomes of agricultural management practices (Hartmann et al. 2015). The soil bacterial community is shaped by multiple interacting factors, including plant genetics driving root traits (Escudero‐Martinez et al. 2022), temporal species succession (Hannula et al. 2019), physico‐chemical soil properties (Sánchez‐Marañón et al. 2017) or geographical soil origin (Coller et al. 2019). The fungal soil community is determined by the plant rhizosphere effect and plant species‐specific effects (Hannula et al. 2019). Furthermore, while fungi provide labile carbon resources for bacteria (Wang and Kuzyakov 2024), both microbial groups drive nutrient transformations and serve as a primary food source for higher trophic levels, such as microarthropods (Gorban et al. 2019). Soil protists can be divided into consumers, phototrophs and parasites. As shown in experimental microbial microcosms, protists feeding on microorganisms modulate the community stability and coexistence among prey species (Karakoç et al. 2020). In vegetable‐planted soils, organic fertilization increases the abundance of pathogenic protists (Lin et al. 2023). Meanwhile, phototrophic protists exhibit a wider niche breadth than the other two functional groups and consumers represent the most diverse group (Lin et al. 2023). Therefore, key soil functions such as biogeochemical cycling, water regulation, pollution mitigation and agricultural biomass production rely heavily on overall soil biodiversity and its complex interactions (Tibbett et al. 2020).

Soil biodiversity in agricultural lands is continuously exposed to a variety of stressors such as pesticide and fertilizer applications and soil disturbance (Orgiazzi et al. 2016). Often these agricultural practices lead to an agricultural soil microbiome that is different from that of more natural lands (Creamer et al. 2016). Agricultural practices such as the application of mineral fertilizers lead to a decline of soil organic matter (SOM), can significantly reduce soil pH, accelerate soil acidification and affect the nitrogen cycle and the diversity and composition of the microbial community (Ouyang and Norton 2020).

Soil DNA metabarcoding via High‐Throughput Sequencing serves as a robust approach for assessing microbial biodiversity in agricultural systems (Kestel et al. 2022). Among major fruit crops, apple ranks second in global production volume behind banana (O'Rourke 2021), making it a key component of the agricultural landscape. However, DNA‐based biodiversity studies in multi‐year crop systems such as apple orchards remain limited (Deakin et al. 2018; Tian et al. 2021; Hulsmans et al. 2022) and little is known about protist diversity in these soils (Oliverio et al. 2020; Lori et al. 2025). This lack of a comprehensive overview on soil biodiversity in managed apple orchards represents a significant knowledge gap regarding perennial agroecosystems.

This study used meta‐barcoding of prokaryotes, protists and fungi in relation to physico‐chemical soil parameters to gain a holistic understanding on biodiversity and its driving factors in 26 different apple orchards of a regional growers' association in Northern Italy where approximately 20% of Italian apples are produced. Based on the premise that standard orchard management generates spatial variation between orchards driven by local site conditions (Deakin et al. 2018), we tested the following hypotheses: prokaryotic, fungal and protistan communities respond differently to abiotic soil characteristics despite being subject to uniform agricultural management of the same growers' association (H1). Site‐specific physico‐chemical soil parameters account for a greater proportion of the variation in microbial community composition than geographic distance/dispersal (H2). Furthermore, we provided baseline information on microbial diversity in Italian apple orchards, a prerequisite to understanding changes related to time and management practices. Reaching beneficial outcomes for productivity requires knowledge of how soil communities are assembled across taxonomic groups and spatial scales (Larsen et al. 2023).

## 2 Materials and Methods

### 2.1 Sampling Sites

Twenty‐six apple orchards (ID from 1 to 26) of a regional growers' association (Associazione Produttori Ortofrutticoli Trentini—APOT: apple growing area > 8000 ha and approximately 4000 associated farmers) in the Trentino region (Northern‐Italy; Figure S1) were sampled between the tree‐rows in summer 2023. These orchards represented the biological replicates across the study region and were representative of conventional management practices of APOT. Sampled orchards covered a restricted elevation gradient (Table 1) as a proxy for air temperature (i.e., air temperature decreases with increasing altitude). Soil was taken from the upper 20 cm using a manual, one‐piece 7‐cm diameter auger (Eijkelkamp, Edelman model) and the top 5 cm of soil were discarded to focus exclusively on the mineral soil layer directly influencing the rhizosphere. To account for micro‐spatial soil heterogeneity, four individual soil cores were collected per site and homogenised in a plastic bag to form a single composite sample. Small‐scale (1–5 m) spatial variability in apple orchards is random (Deakin et al. 2018) and was therefore not considered. During sampling, soil samples were stored at 6 to 8°C.

**TABLE 1:** Chemical and physical soil variables; one standard deviation (sd), maximum (max), minimum (min), soil organic matter (SOM).

Environmental variable	Mean/median	SD	Minimum	Maximum
Altitude (m a.s.l.)	593.2/639.0	236.1	79.0	999.0
Sand (g kg^−1^)	383.3/394.0	148.4	120.0	776.0
Silt (g kg^−1^)	479.7/463.0	104.2	194.0	640.0
Clay (g kg^−1^)	136.9/125.0	66.0	30.0	270.0
pH	7.8/7.9	0.2	7.1	8.1
Total CaCO_3_ (g kg^−1^)	215.0/187.0	162.8	11.0	557.0
Active CaCO_3_ (g kg^−1^)	44.7/20.0	42.4	10.0	124.0
Conductibility (μS cm^−1^)	137.7/138.0	27.6	92.0	203.0
SOM (g kg^−1^)	57.0/54.5	15.9	27.0	90.0
N_tot_ (g kg^−1^)	3.3/3.1	1.0	1.5	5.6
P (mg kg^−1^)	49.1/43.5	29.1	6.0	122.0
K (mg kg^−1^)	182.7/165.0	82.8	84.0	459.0
Mg (mg kg^−1^)	419.0/345.0	280.7	140.0	1319.0
Ca (mg kg^−1^)	6.2/6.8	2.1	2.6	9.4
B (mg kg^−1^)	0.5/0.5	0.2	0.2	1.1
Fe (mg kg^−1^)	47.7/43.4	20.0	17.4	92.4
Mn (mg kg^−1^)	12.1/11.8	4.8	4.2	25.1
Cu (mg kg^−1^)	24.1/13.8	28.3	1.7	121.0
Zn (mg kg^−1^)	9.1/8.0	4.9	0.5	19.6
Cd (mg kg^−1^)	0.1/0.1	0.0	0.1	0.2
Pb (mg kg^−1^)	4.2/3.6	2.6	1.2	13.6

### 2.2 DNA Extraction, Sequencing and Analysis of rRNA Gene Sequences

Each sample was sieved (mesh size: 0.2 mm) and stored at −80°C until DNA extraction. Total DNA was extracted from 0.25 g of homogenized soil using the PowerSoil DNA isolation kit (MO BIO Laboratories Inc., CA, USA). This approach ensures that each analysed subsample is representative of soil biodiversity (Kirse et al. 2021; Taberlet et al. 2012). DNA quality and quantity were assessed using a NanoDrop spectrophotometer prior to downstream analyses. eDNA was then amplified using primers for prokaryotes (16S), protists (18S) and fungi (ITS; Table S1). Positive and negative controls were included in all PCR runs to ensure the reliability and accuracy of the amplification and the resulting PCR products were verified via agarose gel electrophoresis. Finally, the amplicon libraries were pooled in equimolar amounts and sequenced on the Illumina MiSeq platform using a 2 × 300 bp paired‐end (PE300) sequencing strategy.

Amplicon sequence variants (ASVs) were obtained using package DADA2 (Callahan et al. 2016, 2017) using the following settings: truncation length: 250, 250; trimLeft: 19, 20 for 16S; trimLeft: 20, 21 for 18S; maxEE: 2, 5 for 16S, 18S and ITS; specifically for ITS: minLen: 50; maxN: 0; truncQ: 2. ASVs were classified using the RDP classifier (Wang et al. 2007) with the SILVA (Quast et al. 2013) reference version 138 set for prokaryotes, PR2 (Guillou et al. 2013) version 5.0.0 for protists and UNITE version 9.0 (Nilsson et al. 2019; Abarenkov et al. 2024) for fungal ITS. The bootstrap cut‐off value in RDP was 95 for prokaryotes and 80 for protists and fungi. While for prokaryotic communities the 95 cut‐off is essential to maintain high specificity and prevent the misassignment of novel, uncharacterized prokaryotic taxa to known genera (Lan et al. 2012), a 80% cutoff is widely used in fungal (e.g., Yan et al. 2022; Lavallee et al. 2024) and protistan studies (e.g., Salmaso et al. 2020; Stern et al. 2023), providing a conservative, high‐confidence compromise that avoids false positives without over‐filtering true fungal and protistan taxa. ASVs that were not identified at a kingdom/supergroup level were removed from the dataset because of uncertain taxonomic status (e.g., Fungi incertae sedis). PR2 includes nine taxonomic ranks and since eukaryotic taxonomy is constantly evolving, we focused on phylum (division), class and genus level. For analyses, the ASV table was rarefied to an even depth specific for each primer (Table S2) as it is appropriate for eDNA (Schloss 2024). Success of taxonomic assignment at genus level was highest for protists and lowest for bacteria (Table S2).

All soil samples for prokaryotes were successfully sequenced. For protists, one soil sample (ID1) was removed because of low read number (153 reads). For fungi, sequencing of two samples failed (ID9, ID15); five samples (ID2; ID10, ID20; ID19, ID23) were discarded from analyses because of low read number (70 reads) and thus 19 samples remained.

### 2.3 Soil Variables

Physico‐chemical analyses were carried out on the air‐dried fine soil fraction (< 2 mm). Soil texture was determined as the amount of sand (2 mm–50 μm), silt (50 μm–2 μm) and clay (< 2 μm) by wet sieving and a hydrometric assay of soil dispersant solution (sodium hexametaphosphate). pH was measured in a soil–water suspension (w/v 1:2.5). Total carbonates were determined on powdered samples using the volumetric method, measuring CO_2_ produced after the addition of hydrochloric acid (ISO 10693: 1995). The SOM was assessed as the difference between total carbonates and total carbon, measured using Dumas dry combustion of powdered soil and a thermal conductivity detector (ISO 10694: 1995) and using an organic carbon conversion factor of 1.724. Total nitrogen (N_tot_) was measured by dry combustion (ISO 13878: 1998). The available fraction of elements was extracted in a DTPA solution and metals (copper, zinc, iron, manganese, lead) were detected using inductively coupled plasma optical emission spectroscopy (ICP‐OES). The cation exchangeable capacity was determined by ICP‐OES determination of magnesium adsorbed on the soil exchangeable surface after monosaturation with barium and exchange with magnesium added as magnesium sulphate. The exchangeable fractions of magnesium, potassium and calcium were extracted in ammonium acetate (pH 7.00) and detected using ICP‐OES. The assay of assimilable phosphorus was carried out using the Olsen method, providing solubilization of phosphorus in a natrium carbonate solution and determination using spectrophotometry with the ascorbic acid method. Soluble boron was extracted using a hot treatment with a diluted solution of calcium chloride (w/v 1:2) and determined using ICP‐OES. From here onwards, metals and nutrients were abbreviated by their chemical symbols.

### 2.4 Statistical Analyses

We used R version 4.5.1 (R Core Team 2025) for all analyses. In plotting, we used colourblind friendly colours (package cols4all; Tennekes and Puts 2023).

We applied a correlation matrix, the variance inflation factor (VIF) and a principal component analysis (PCA) to inspect dependencies among environmental variables.

#### 2.4.1 Alpha Diversity

Alpha diversity was calculated as Hill numbers (Chao et al. 2024) with package hillR (Li 2018). We assessed which orchard had values below the 25% percentile, above the 75% percentile and between the 25% and 75% percentile of alpha diversity indices to inspect if different organismal groups show the same orchard ranking. Furthermore, we correlated alpha diversity indices with environmental variables (*p*‐values were corrected for multiple testing).

We assessed the core ASVs of soil biodiversity that occurred in all sampled orchards. For prokaryotes, we compared the genera of these core ASVs to the genera that are globally dominant in different soil types (Delgado‐Baquerizo et al. 2020). We also evaluated the soil nutrient status by the ratio between the relative abundance of Proteobacteria (P) and Acidobacteria (A), with a high P/A ratio indicating a copiotrophic status and a low ratio an oligotrophic status (Choudhary et al. 2020).

#### 2.4.2 Prediction of Prokaryotic Functions

We acknowledge that the presence of potential functions is not proof of their active state; nevertheless, predicting these functions is a complementary approach to studying soil biodiversity (Sansupa et al. 2021). Putative functions were predicted with functional annotation of prokaryotic taxa (FAPROTAX—Louca et al. 2016; package microeco—Liu et al. 2021) that assign metabolic functions to prokaryotes based on literature information of cultured taxa and provide functions as percentage.

#### 2.4.3 Annotation of Fungal Genera in Trophic Feeding Modes

We annotated ecological functions to fungal genera (package FUNGuildR; Furneaux and Song 2021). To ensure high confidence, only taxa with a confidence ranking of ‘Highly Probable’ or ‘Probable’ were retained. Functional roles were further parsed into distinct categories, including arbuscular mycorrhizal fungi (AMF), ectomycorrhizal fungi (ECM), ericoid/orchid mycorrhizal fungi, pathotrophs (pathogens/parasites) and saprotrophs (decomposers). Unassigned taxa or those with low confidence scores were classified as unassigned/unknown, representing uncultured environmental fungi whose functional traits have not yet been empirically described.

#### 2.4.4 Beta‐Diversity and Distance Decay Relationships

The similarity among pairs of biological communities typically decays with increasing spatial and/or environmental distance (Graco‐Roza et al. 2022). This pattern stems from dispersal limitation related to physical barriers and dispersal ability (Hubbell 2004) and species‐specific responses to spatially structured environmental variation (Cottenie 2005). We applied the Aitchison distance (Euclidean distance on centred log‐ratio‐transformed values) to the rarefied ASV matrices to account for the log‐ratio structure of the data and give equal weight to relative changes in both high‐ and low‐abundance taxa. We assessed differences in the mean beta‐diversity (Aitchison distance) between organisms by non‐parametric one‐way ANOVA and post hoc comparisons with Benjamini‐Hochberg correction for multiple testing. Furthermore, using linear regression and Mantel correlation, we evaluated the distance‐decay relationship of beta‐diversity (Aitchison distance) with both orchard spatial distance and environmental distance (Euclidean distance between standardized environmental variables).

#### 2.4.5 Community Assembly Processes

We applied the normalized stochasticity ratio that shows good performance at regional scale (NST; Ning et al. 2019) to assess the relative contribution of stochastic versus deterministic assembly processes. NST was calculated based on the Ružička metric using a null model (1000 permutations) where the probabilities of taxa occurrence are proportional to the observed occurrence frequencies and the taxon richness in each sample is fixed as observed. Deterministic processes dominate with NST < 50% and stochastic processes with NST > 50% (Ning et al. 2019). We also estimated the overall contribution of neutral dynamics to community assembly using Sloan's neutral modelling framework (neutral community model; Sloan et al. 2006) that assumes that communities assemble primarily through stochastic demographic processes, such as random dispersal and ecological drift, with minimal influence from selective environmental pressures. A low immigration rate m corresponds to a restricted dispersal of taxa while m = 1 implies that each local community is simply a random sample from the overall metacommunity.

Finally, we partitioned beta‐diversity into the two antithetic processes of community assembly, namely taxa turnover and nestedness using package betapart (Baselga et al. 2025). We used both presence/absence and abundance data and compared results because eDNA abundance data is semi‐quantitative and measured with bias.

#### 2.4.6 Niche Breadth

We applied Levin's measure of niche breadth (package EcoUtil; Salazar 2025). A high niche breadth indicates that an ASV occurs widely and evenly along a wide range of sites, resulting in a wider niche breadth. We tested niche breadth differences for generalists by non‐parametric one‐way ANOVA and post hoc pair‐wise comparisons.

#### 2.4.7 Disentangling the Effect of Space and Environment on Communities

We investigated the importance of environment and space on ASV diversity by applying variance partitioning (package vegan; Oksanen et al. 2024). As spatial predictors, we used distance‐based Moran's Eigenvector Maps (dbMEMs), which are linearly independent spatial descriptors that allow for modelling spatial structures over a wide range of spatial scales (Dray et al. 2006). Only positive spatial dbMEMs, accounting for positive spatial correlation and explaining the highest variability of community data, were kept (package adespatial; Dray et al. 2025). As environmental variables, we used scaled variables that explained the highest variability in a redundancy analysis (RDA) with the Hellinger‐transformed community matrix of ASVs abundances. Variables with VIF > 10 were excluded. We applied forward selection of variables based on adjusted *R* ^2^ (package vegan; Oksanen et al. 2024) and only used ASVs that were present in at least 50% of samples. Models and ordination axes were tested for significance using 999 permutations of the community data. Finally, we applied variance partitioning with selected environmental and spatial variables.

## 3 Results

### 3.1 Chemical and Physical Soil Variables

Chemical variables showed considerable heterogeneity across the investigated orchards (Table 1). Heavy metals such as Cu, Zn and Pb varied by up to 71‐, 39‐ and 11‐fold, respectively, and remained generally below the regulatory threshold levels for agricultural soils (Tóth et al. 2016). High variability was also observed for total CaCO_3_ (51‐fold), active CaCO_3_ (12‐fold) and P (20‐fold), whereas the remaining variables displayed a less pronounced variation (< 10‐fold; Table 1).

Many environmental variables were correlated (Figure S2). We kept clay, pH, conductivity, Mg, K, N_tot_, B, Zn, Fe, Mn, Cu, Cd, Pb, year and altitude (all VIFs < 10) in multivariate analyses. In PCA, orchards were separated mainly based on heavy metals (negative loadings on PCA1; Figure 1) and soil texture, conductivity and pH (positive loadings on PCA1). Orchards ID1, 3, 23 and 24 showed the most extreme environmental conditions (Figure 1).

**FIGURE 1:**
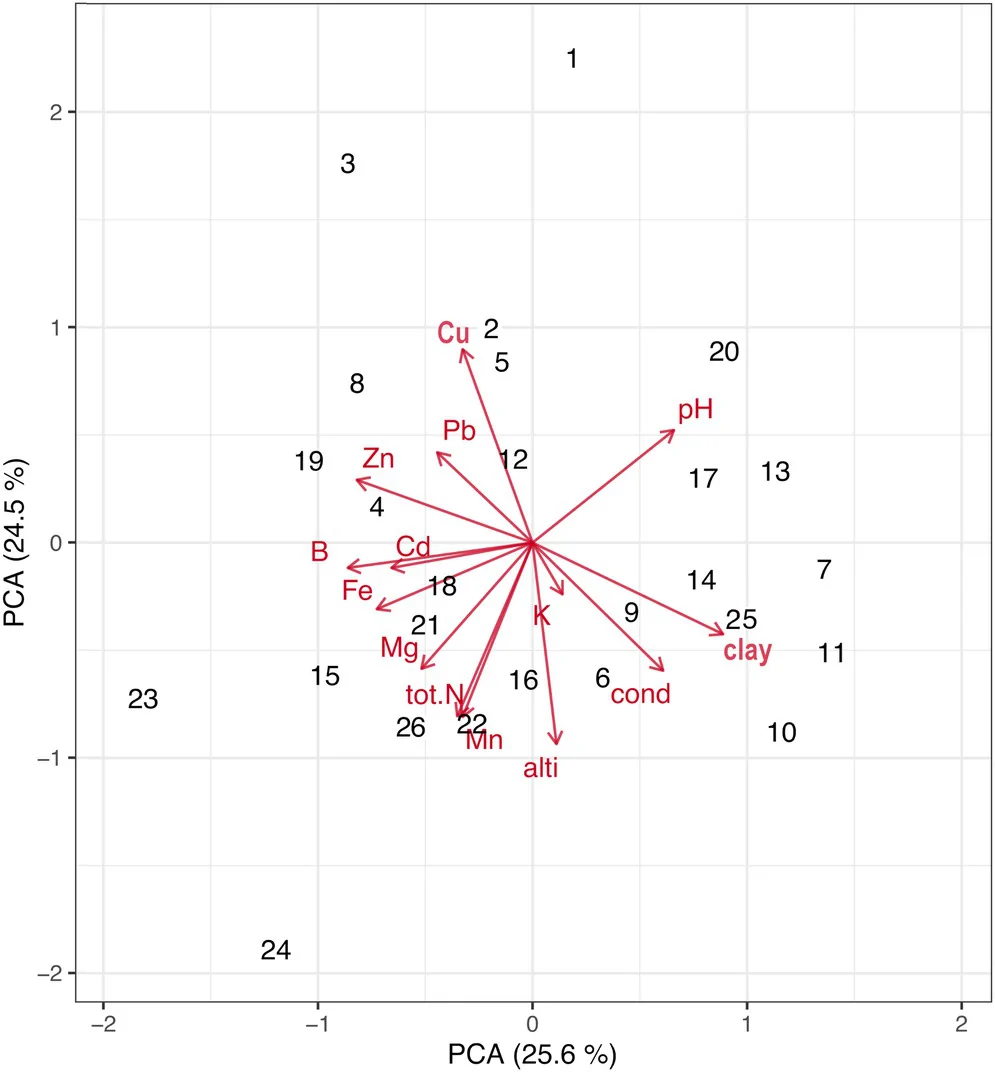
PCA (in parentheses explained variation) with standardized environmental soil variables (VIF < 10) of the 26 sampled apple orchards. Numbers indicate orchard IDs; conductivity (cond), altitude (alti), total nitrogen (tot.N).

### 3.2 Alpha Diversity and Orchard Grouping

Of the total number of ASVs found across all orchards, individual orchards harboured only a small fraction of this total pool (prokaryotes < 25%; protists < 13%; fungi < 16%; Table 2) and even fewer ASVs were abundant or dominant (Table 2). A comparison of orchard rankings based on alpha diversity indices showed marked discrepancies across bacteria, protists and fungi, except for orchards ID13 and ID18 (high values; Figure 2). For fungi (Figure 2), orchards with low alpha diversity (< 25% quantile) were characterised by elevated heavy metal concentrations (IDs 1, 3, 4, 5, 26; Figure 1). Only orchard ID26 consistently ranked lowest (< 25th percentile) for both richness and Shannon diversity of bacteria and fungi (Figure 2).

**TABLE 2:** Alpha diversity indices (ASV richness, Shannon diversity, inverse Simpson diversity) of prokaryotes, protists and fungi.

		Prokaryotes	Protists	Fungi
Total number of ASVs		11,987	8552	6538
ASV richness	Mean ± SD	1684 ± 462	813 ± 151	756 ± 229
	Range	929–2953	480–1100	113–1012
	25th percentile	1477	739	699
	75th percentile	1790	924	915
Abundant ASVs (Shannon div.)	Mean ± SD	738 ± 226	293 ± 90	130 ± 53
	Range	385–1397	79–495	37–205
	25th percentile	599	230	90
	75th percentile	839	349	185
Dominant ASVs (inverse Simpson div.)	Mean ± SD	252 ± 137	111 ± 52	46 ± 22.1
	range	93–667	9–241	13–83
	25th percentile	164	73	30
	75th percentile	285	135	63

*Note:* The 25th and 75th percentiles are used to rank orchards (Figure 1).

Abbreviations: div, diversity; SD, standard deviation.

**FIGURE 2:**
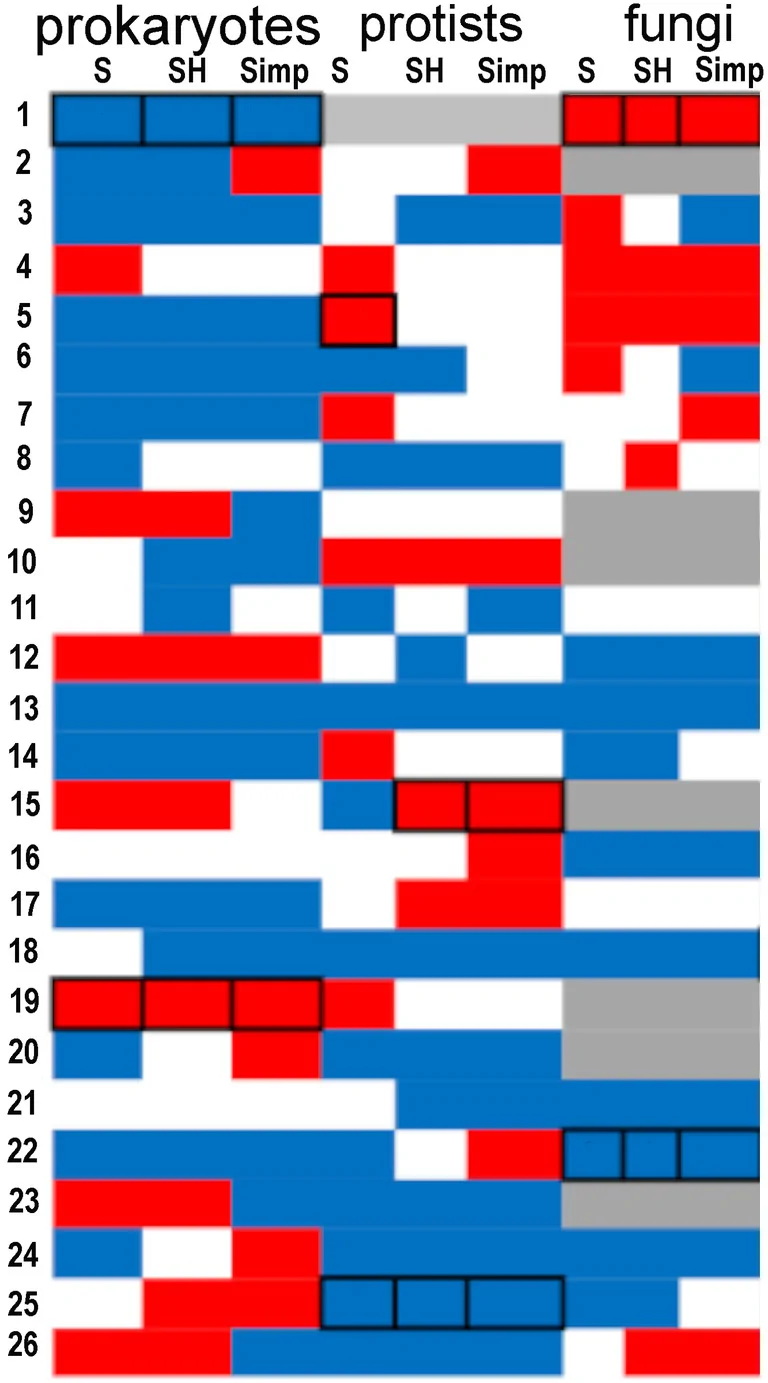
Ranking of orchards based on alpha diversity (S, ASV richness; SH, Shannon diversity; Simp, inverse Simpson diversity). Colour coding: red cells indicate values in the lower quartile (< 25th percentile), blue cells indicate values in the upper quartile (> 75th percentile) and white cells represent the interquartile range (25th–75th percentile); grey cells indicate missing orchards. Framed boxes indicate highest (blue) or lowest (red) observed values.

Regarding the correlation between alpha diversity and environmental variables (Table 3), fungal ASV richness increased with altitude, SOM content and N_tot_, while it decreased markedly with Cu. In contrast, prokaryotic ASV richness decreased with altitude. The number of the most abundant fungal ASVs increased with SOM and N_tot_ (Table 3), similarly to fungal ASV richness.

**TABLE 3:** Pearson's correlation corrected for multiple testing between Hill numbers (S, ASV richness; Simp, inverse Simpson diversity) and environmental variables including age; protist‐based alpha diversity indices and Shannon diversity did not show any significant correlations; soil organic matter (SOM).

Hill	Variable	Prokaryotes	Fungi
S	Altitude	−0.59*	0.63*
	SOM		0.60*
	N_tot_		0.59*
	Cu		−0.73**
Simp	SOM		0.62*
	N_tot_		0.63*

* *p* < 0.05.

** *p* < 0.01.

### 3.3 Bacteria and Archaea

Prokaryotic ASVs were assigned to 42 phyla, with the 10 most abundant phyla accounting for 89%–97% of the total relative abundance (Figure 3). Phyla Actinobacteriota, Acidobacteriota and Proteobacteria were the dominant phyla in 15 orchards, Actinobacteriota, Crenarchaeota and Proteobacteria dominated in 6 orchards and Actinobacteriota, Acidobacteriota and Crenarchaeota in 5 orchards (Figure 3; Table S3). Crenarchaeota, the only archaeal phylum detected, ranged from 3.4% (ID1) to 21.8% (ID12) of relative abundance (overall mean ±1 standard deviation across orchards: 14% ± 4.8%).

**FIGURE 3:**
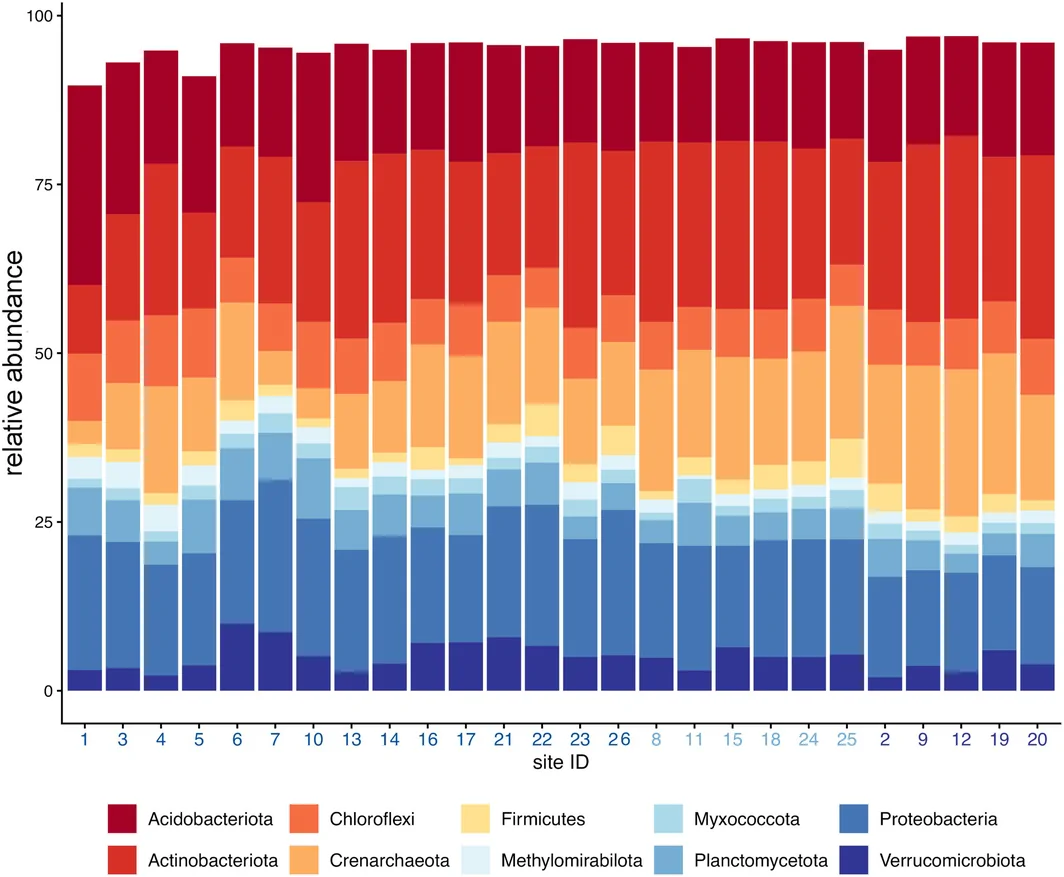
Relative abundance of the 10 most abundant prokaryotic phyla across orchards. Orchards are ordered according to the relative abundance of the three most dominant phyla. From left to right: Group 1: Actinobacteriota, Acidobacteriota and Proteobacteria (*n* = 15); Group 2: Actinobacteriota, Crenarchaeota and Proteobacteria (*n* = 6); Group 3: Actinobacteriota, Acidobacteriota and Crenarchaeota (*n* = 5); Table S3. Numbers are orchard IDs.

The P/A ratio of orchards ranged from 0.6 (ID1) to 1.41 (ID7) (mean: 1 ± 0.19).

Only 0.65% of prokaryotic ASVs constituted the core community of investigated apple orchards. These core ASVs (*n* = 78) belonged primarily to phyla Acidobacteriota (*n* = 22), Proteobacteria (*n* = 21), Actinobacteriota (*n* = 14) and Crenarchaeota (*n* = 5), with the remaining 16 ASVs assigned to other phyla (Tables S4 and S5). Collectively, they accounted for 10% (ID1) to 25% (IDs 8, 12, 19) of relative abundance (20% ± 3.4% across orchards; Figure 4). Among these core ASVs, ASVs assigned to the genera *Gaiella*, *Pedomicrobium*, *Pseudarthrobacter* and *Candidus Xiphinematobacter*, the families Vicinamibacteraceae and Methyloligellaceae, orders Vicinamibacterales and Rhizobiales, as well as unclassified bacteria, had a relative abundance > 1% (Figure 4). Of these core ASVs, *Bradyrhizobium*, *Cellulomonas*, *Iamia*, *Nocardioides*, *Pedomicrobium*, *Steroidobacter* (2 ASVs) and *Skermanella* belonged to the genera of globally occurring soils phylotypes (Delgado‐Baquerizo et al. 2020; Table S5).

**FIGURE 4:**
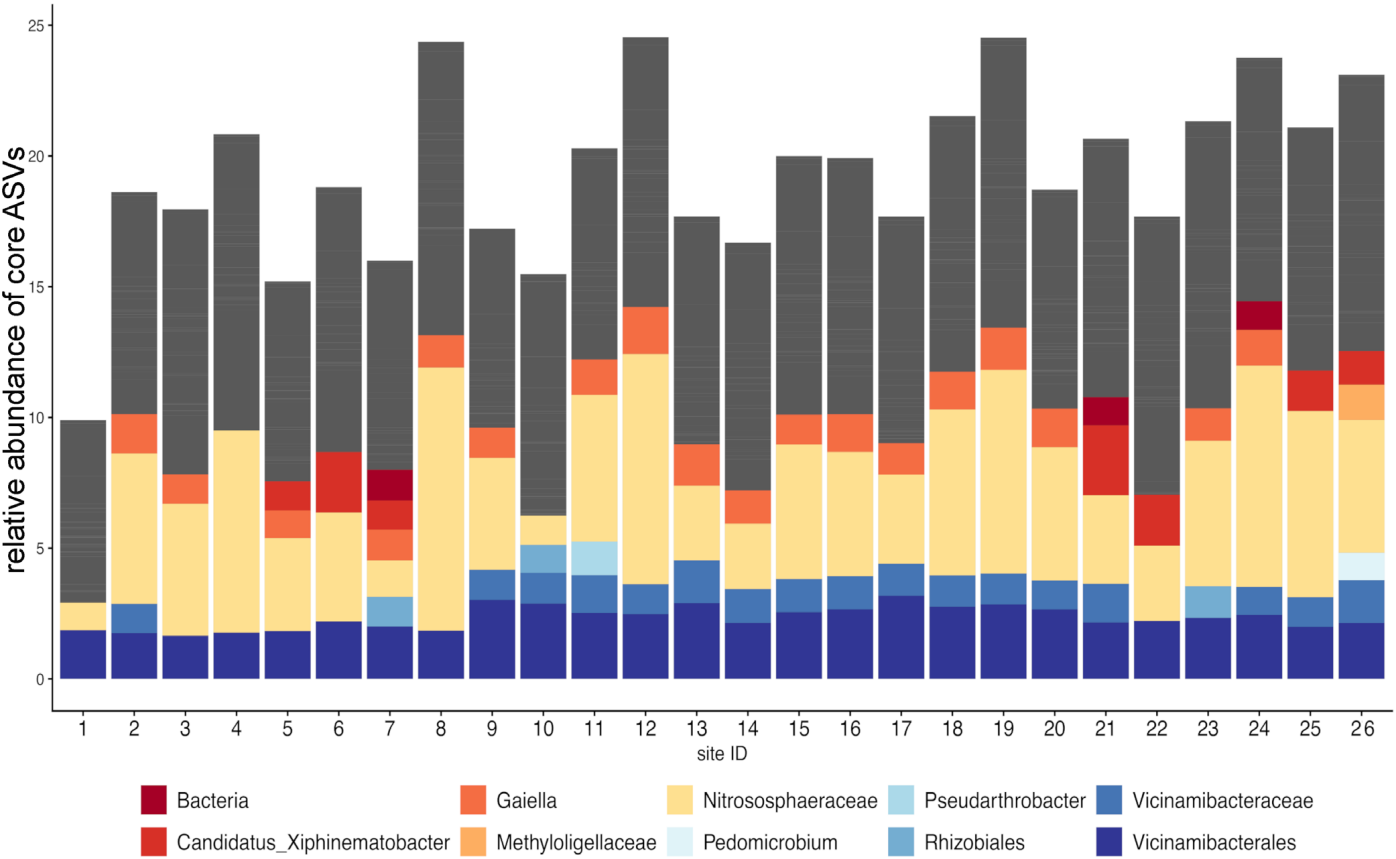
Total relative abundance of the core prokaryotic ASVs occurring in all orchards. Numbers are orchard IDs. Only ASVs with abundance > 1% are shown. The grey bar is the relative abundance of core ASVs < 1%. Reported is the highest level of taxonomic assignment.

The number of unique prokaryotic ASVs ranged from 38 (ID19) to 1360 (ID1) ASVs per orchard (overall mean: 240 ± 273 unique ASVs per orchard). Together, these unique ASVs accounted for 1% (ID19) to 21% (ID1) of relative abundance (overall mean: 5% ± 4.6%). Phyla representing at least 10% of the relative abundance of unique ASVs per orchard belonged primarily to Actinobacteriota, Planctomycetota and Proteobacteria. Additionally, in some orchards (IDs 1, 2, 4, 7, 10, 14, 16, 18, 19, 25) they also included Acidobacteriota, Chloroflexi, Firmicutes and Myxococcota.

Fifty‐two functions were predicted with FAPROTAX based on ASV occurrence. Six of these functions represented at least 5% of the functional abundance in at least one sample (Figure 5). Of these functions, aerobic ammonia oxidation (15% ± 4.6%), nitrification (15% ± 4.6%) and aerobic chemoheterotrophy (15% ± 2.3%) accounted for the highest mean abundances across all orchards. Anaerobic chemoheterotrophy (13% ± 1.5%), dark hydrogen oxidation (3% ± 0.9%) and animal parasites and symbionts (2% ± 1.2%) were less abundant. Orchard ID1 showed the lowest relative abundance for aerobic ammonia oxidation (4.6%) and nitrification (4.6%), but the highest for anaerobic chemoheterotrophy (17.6%). Orchard ID11 exhibited the lowest abundance for animal parasites and symbionts (0.5%) and the highest for aerobic chemoheterotrophy (20.7%). Orchard ID5 showed the lowest abundance for aerobic chemoheterotrophy (10.9%) and dark hydrogen oxidation (1.4%), whereas orchard ID23 showed the highest abundance for dark hydrogen oxidation (5.4%).

**FIGURE 5:**
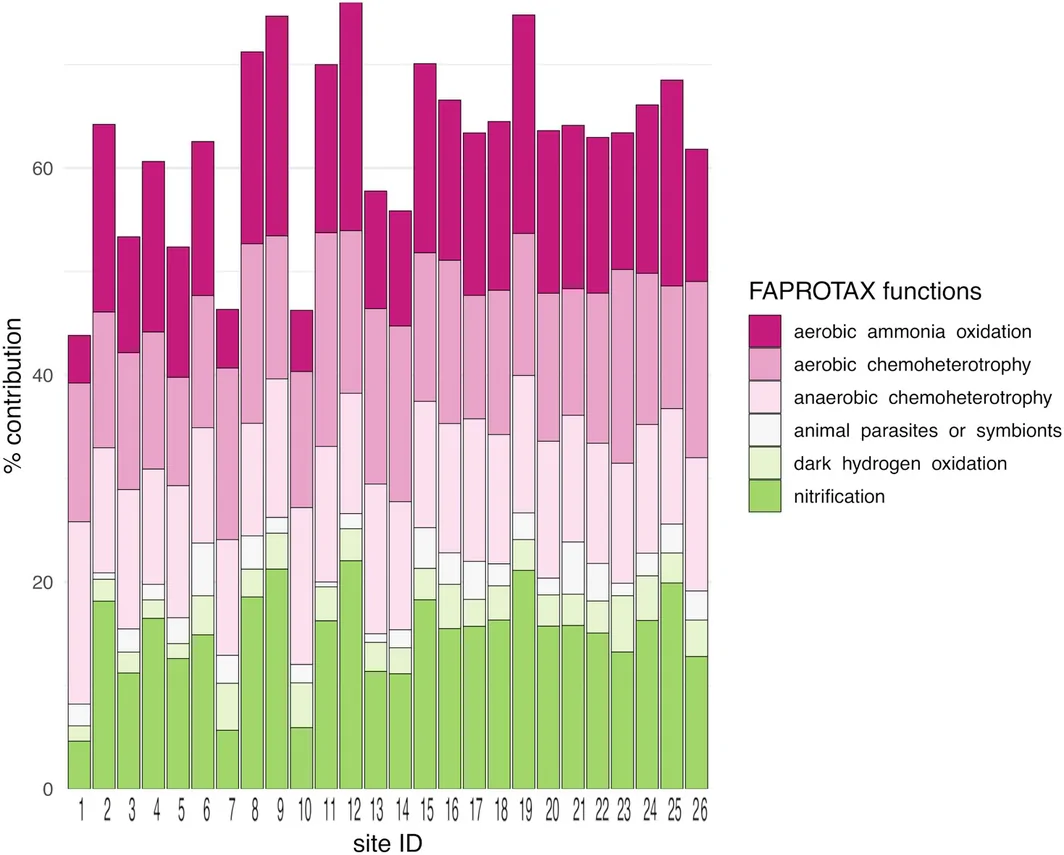
Predicted prokaryotic functions with FAPROTAX that accounted for at least 5% of relative abundance. Numbers are orchard IDs.

### 3.4 Protists

Protist ASVs were assigned to 22 phyla, with the 10 most abundant accounting for 95%–99% of the total relative abundance (Figure 6). Alveolata occurred in all orchards accounting for 8% (ID5) to 61% (ID15) of relative abundance. The three most abundant phyla were Alveolata, Evosea and Rhizaria in 14 orchards; Alveolata, Chlorophyta and Evosea in five orchards; and Alveolata, Chlorophyta and Rhizaria in three orchards (Figure 6; Table S6).

**FIGURE 6:**
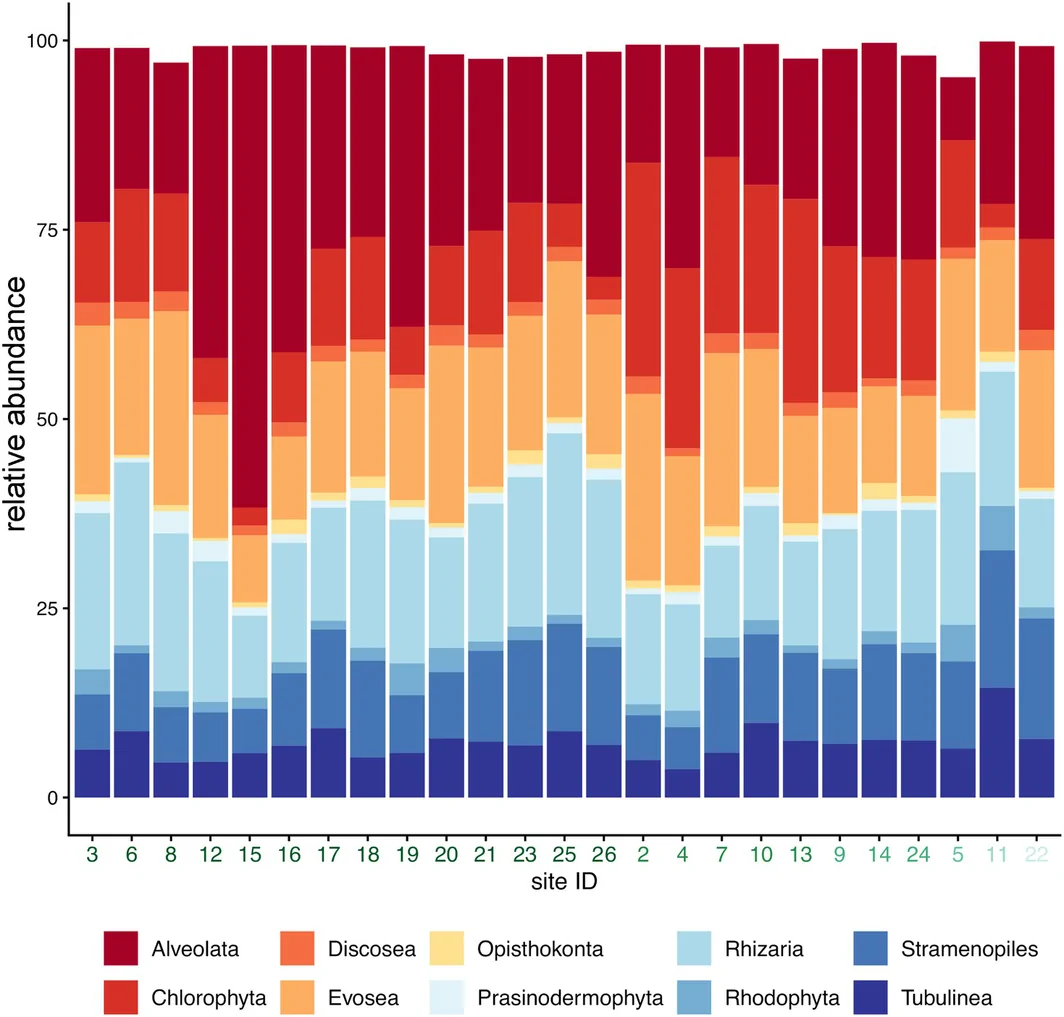
Relative abundance of the 10 most abundant protist phyla. Orchards are ordered from left to right according to the relative abundance of the three most abundant phyla (Group 1: Alveolata, Evosea and Rhizaria [the first 14 sites]; Group 2: Alveolata, Chlorophyta and Evosea [the next five sites]; Group 3: Alveolata, Chlorophyta and Rhizaria [the next three sites]; Group 4: Chlorophyta, Evosea, Rhizaria [ID5]; Group 5: Alveolata, Chlorophyta, Evosea [ID11]; Group 6: Alveolata, Evoseas, Stramenopiles [ID22]; Table S7); groups are indicated by different colours. Numbers are orchard IDs.

Only 0.11% of protist ASVs (*n* = 9; Figure 7; Table S8) constituted the core community. These core ASVs belonged to phyla Evosea (*n* = 3; class Variosea), Stramenopiles (*n* = 3, classes Peronosporomycetes, Eustigmatophyceae), Chlorophyta (*n* = 2; class Chlorophyceae) and Rhizaria (*n* = 1; class Filosa‐Sarcomonadea). Collectively, these few core ASVs accounted for 4% (ID15) to 17% (ID17) of relative abundance (overall mean: 10% ± 3.3% per orchard).

**FIGURE 7:**
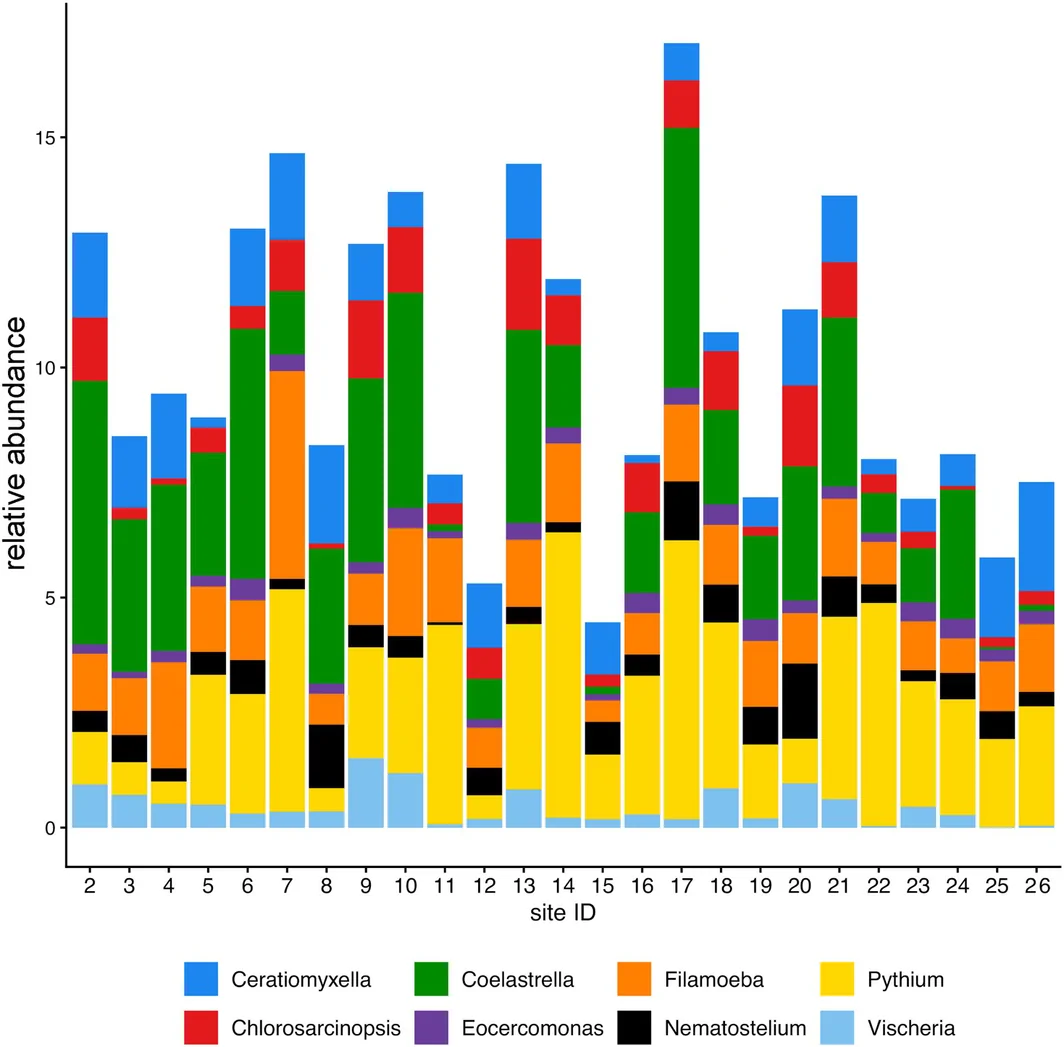
Relative abundance of the core protist ASVs (only genus level was taxonomically assigned). Numbers are orchard IDs. Of the nine core ASVs, two belonged to genus *Pythium*.

The number of unique ASVs ranged from 127 (ID4) to 355 (ID23) ASVs (overall mean: 223 ± 68 unique ASVs per orchard). Together, they accounted for 5% (ID15) to 32% (ID5) of total relative abundance (overall mean: 11% ± 6.2%). Phyla representing at least 10% of relative abundance of unique ASVs belonged mostly to Alveolata, Evosea, Rhizaria and Stramenopiles and in specific orchards to Tubulinea, Rhodophyta, Discoba and Discosea (Table S9).

### 3.5 Fungi

Fungal ASVs were assigned to 19 fungal phyla, with the 10 most abundant collectively accounting for 99%–100% of total relative abundance (Figure 8). Ascomycota, Basidiomycota and Mortierellomycota were the dominant phyla in 17 orchards, whereas Ascomycota, Basidiomycota and Rozellomycota were the most abundant in two orchards (ID1, ID4) (Figure 8; Table S10).

**FIGURE 8:**
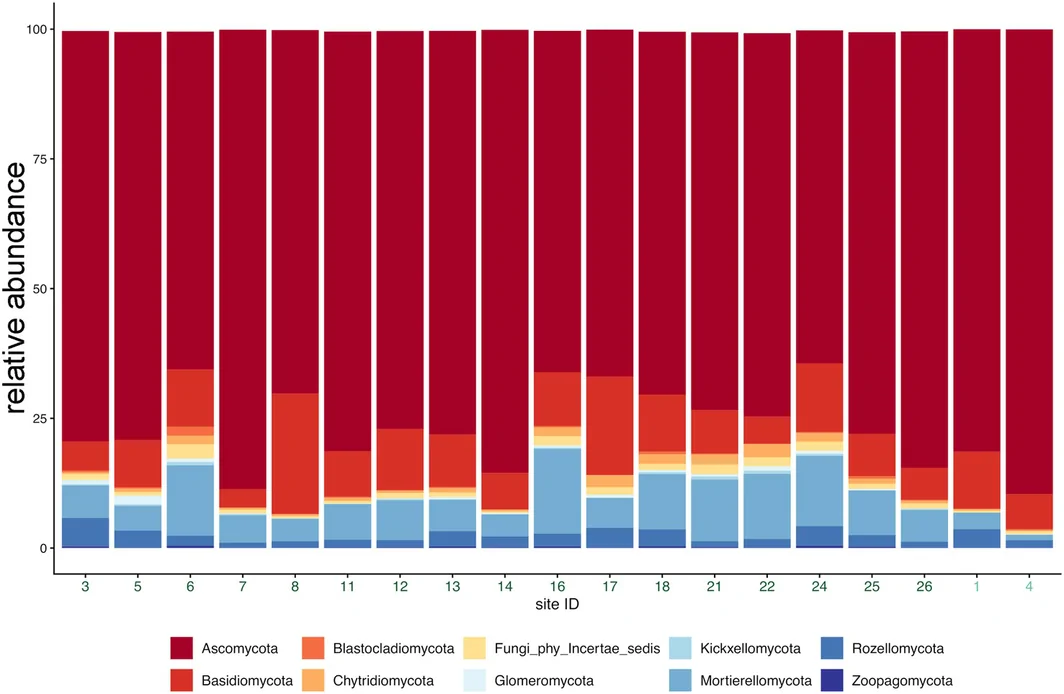
Relative abundance of the 10 most abundant fungal phyla in orchard sites. Orchards are ordered from left to right according to the relative abundance of the three most abundant phyla (Group 1: Ascomycota, Basidiomycota and Mortierellomycota [the first 17 sites]; Group 2: Ascomycota, Basidiomycota and Rozellomycota [the last 2 sites]); groups are indicated by different colours. Numbers are orchard IDs.

Only 0.19% of fungal ASVs (*n* = 13) represented the core fungal diversity. These core ASVs belonged mainly to phyla Ascomycota (*n* = 11), Basidiomycota (*n* = 1) and Mortierellomycota (*n* = 1; Table S11). Collectively, these few core ASVs accounted for 10% (ID25) to 26% (IDs 13, 17) of relative abundance (Figure 9; overall mean: 19% ± 4.4%).

**FIGURE 9:**
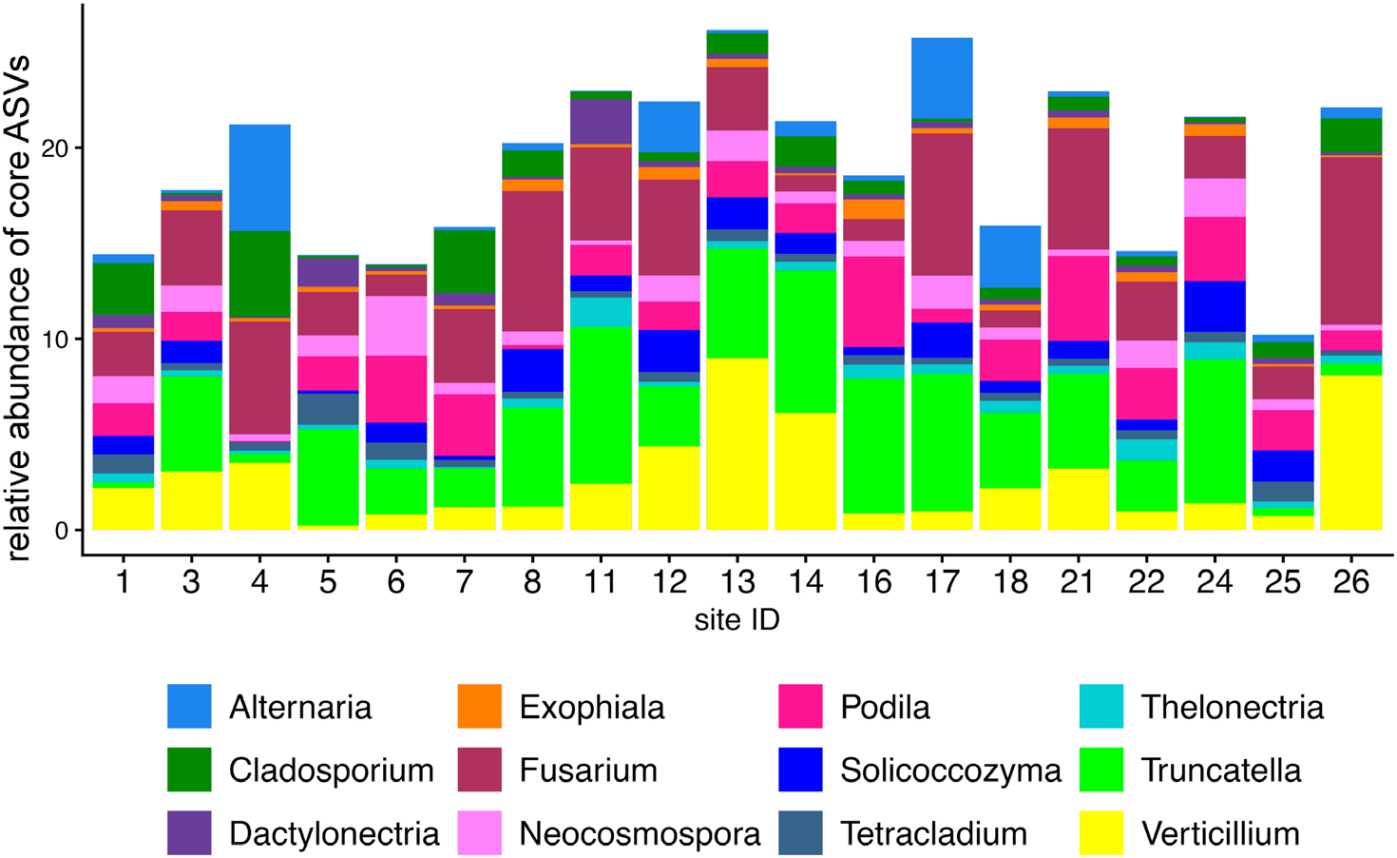
Relative abundance of the core fungal ASVs at genus level in orchards. Of the 13 core ASVs, two belonged to genus *Fusarium*. Numbers are orchard IDs.

The number of unique ASVs ranged from 29 (ID1) to 319 (ID25) ASVs (overall mean: 223% ± 74% unique ASVs per orchard) that collectively accounted for 4% (ID14) to 20% (ID7) of relative abundance (overall mean: 7% ± 4.3%). Phyla accounting for at least 10% of relative abundance of unique ASVs per orchard mostly belonged to Ascomycota, Basidiomycota and Chytridiomycota (Table S12).

Among functional guilds assigned with FUNGuild, many reads remained unassigned (46% ± 5.9%), while saprotrophs (28% ± 6.5%) and pathotrophs (25% ± 7.6%) were highly abundant (Figure 10A). Ectomycorrhizal (0.7% ± 2.16%), arbuscular mycorrhizal (0.2% ± 0.19%) and ericoid mycorrhizal fungi (0.01% ± 0.04%) were less abundant (Figure 10B). No mycorrhizal fungi were detected in orchard ID1, whereas the highest abundance was recorded in orchard ID8 (Figure 10B).

**FIGURE 10:**
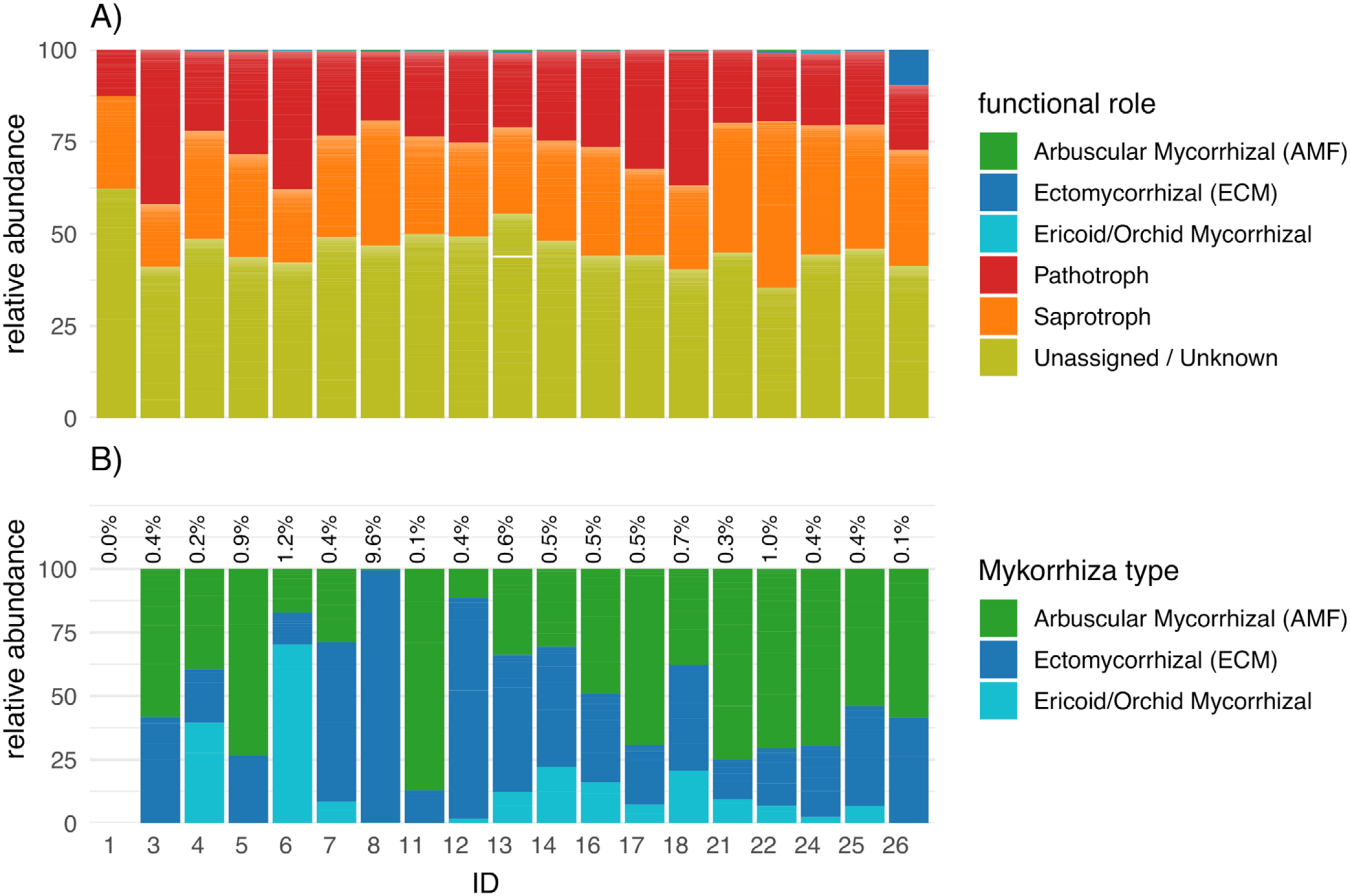
Relative abundance of functional guilds assessed with FUNGuild. (A) Total relative abundance of functional guilds; (B) relative abundance of mycorrhiza. Percentages indicate relative abundance with respect to all functional guilds. Numbers are orchard IDs.

### 3.6 Beta‐Diversity

Mean beta‐diversity between orchards at ASV level was significantly different among organism groups (*p* < 0.001), with protists showing the lowest mean diversity, followed by prokaryotes and fungi (Figure 11A).

**FIGURE 11:**
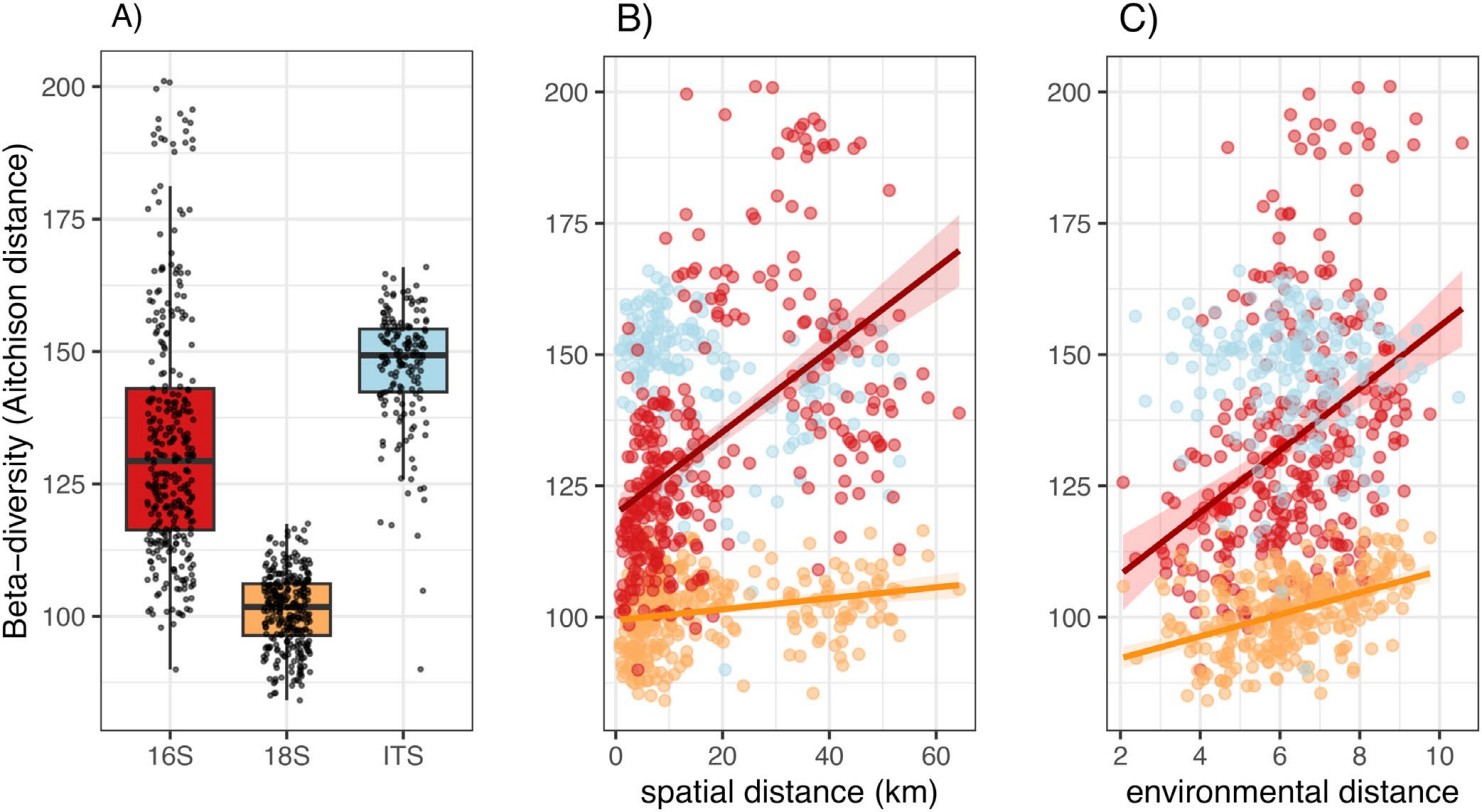
(A) Boxplots of beta‐diversity among orchards for prokaryotes (16S; red), protists (18S; orange) and fungi (ITS; blue). Distance decay relationships of organismal groups with (B) spatial distance and (C) environmental distance. The lighter shaded area is the 95% confidence interval of the exponential decay.

A distance decay relationship with both spatial and environmental distances was found for prokaryotes and protists (Figure 11B,C; Table 4), whereas prokaryotes showed a stronger increase in diversity than protists. Fungal beta‐diversity showed no relationship with spatial nor environmental distance (Figure 11B,C; Table 4).

**TABLE 4:** Mantel correlation and significance level of the relationship of beta‐diversity (Aitchison distance) with both orchard spatial distance and environmental distance (Euclidean distance between standardized environmental factors).

Group	Spatial distance	Environmental distance
	Mantel *r*; sig.	Mantel *r*; sig.
Prokaryotes	0.59***	0.39**
Protists	0.32*	0.45***
Fungi	−0.37^ns^	−0.04^ns^

*** *p* < 0.001.

** *p* < 0.01.

* *p* < 0.05.

^ns^ *p* > 0.05.

### 3.7 Community Assembly Processes

Prokaryotes, protists and fungi showed a predominance of stochastic community assembly (NST_prokaryotes_ = 72.4%; NST_protists_ = 59.5%; NST_fungi_ = 53.5%). Under Sloan's neutral modelling framework, all organismal groups showed low immigration rates close to 0, with prokaryotes (Figure 12A) displaying the highest rate, followed by protists (Figure 12B) and fungi (Figure 12C).

**FIGURE 12:**
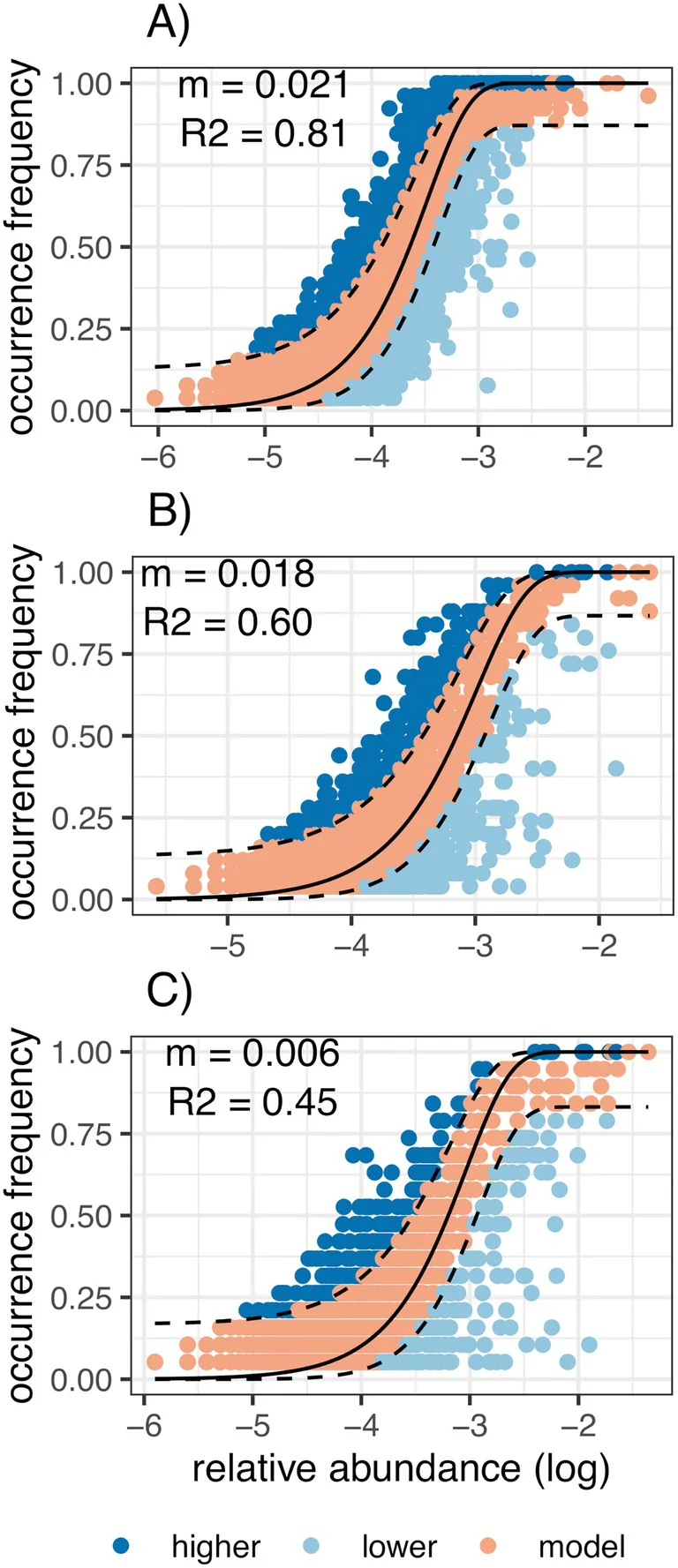
Sloan's neutral modelling framework to assess immigration rates of (A) prokaryotes, (B) protists, (C) fungi; m is the immigration rate and R2 is variability explained.

For both presence/absence and abundance data, replacement of taxa (i.e., turnover/balanced variation in species abundance) dominated for prokaryotes, protists and fungi (> 90%; Table S13).

### 3.8 Niche Breadth

The average niche breadth of generalist ASVs of prokaryotes, protists and fungi differed between each other (*p* < 0.001). Generalists of prokaryotes showed the highest mean niche breadth, followed by protists and fungi (Figure 13).

**FIGURE 13:**
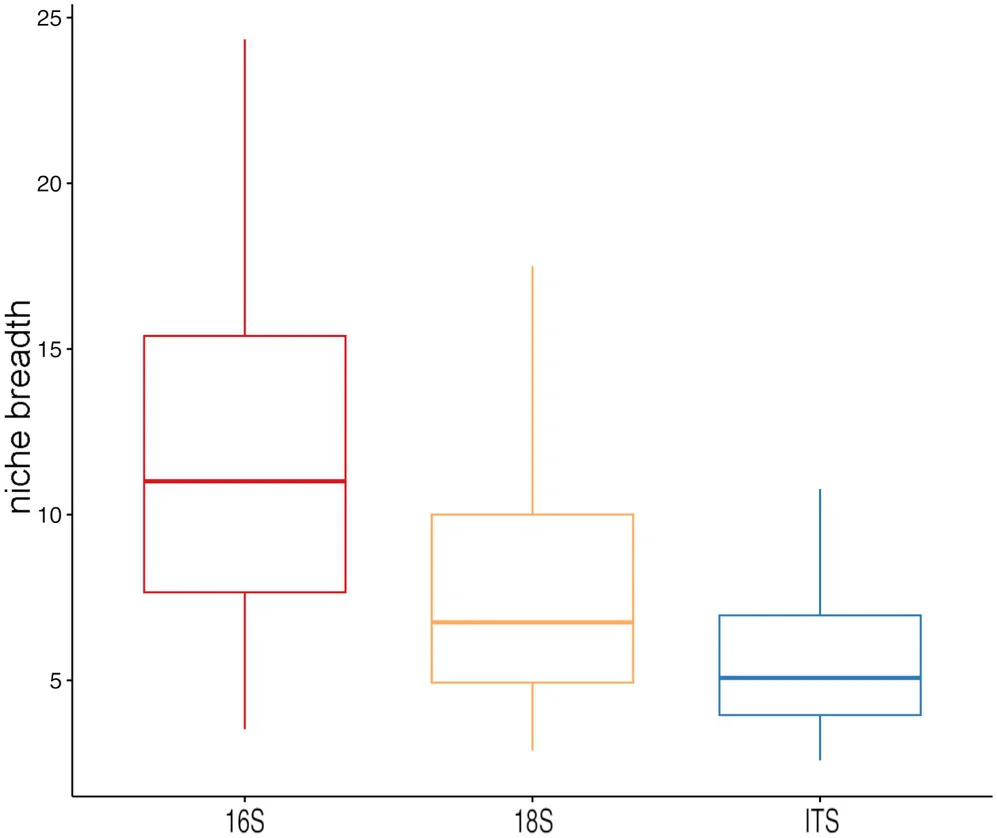
Niche breadth (Levin's B) of generalist ASVs: prokaryotes (16S), protists (18S) and fungi (ITS).

### 3.9 Variance Partitioning

The total explained variation was highest for prokaryotes, followed by fungi and protists (Table 5). Environmental variables explained the greatest proportion of variance in fungi, followed by prokaryotes and protists, whereas spatial predictors explained more variation in bacteria and protists. Three environmental variables influenced multiple groups (altitude, K and Zn were important for both prokaryotes and protists), while others were group‐specific (clay for prokaryotes, age for fungi; Table 5; Figure S3).

**TABLE 5:** Results of variance partitioning of soil communities by environmental and spatial variables.

Predictor	Prokaryotes	Protists	Fungi
Environment			Conductivity
	Clay		
	pH		
	K	K	
	Cu		Cu
	Zn	Zn	
			Age
	Altitude	Altitude	
Space	MEM1, MEM2, MEM4	MEM7, MEM9	
Unique ENV *R* ^2^_adj_	15%***	10%***	17%***
Unique space *R* ^2^_adj_	6%***	1%*	
Shared *R* ^2^_adj_ by space + ENV	9%	5%	
Unexplained *R* ^2^	69%	84%	83%

*Note:* Only significant variables are reported; variability explained adjusted for the number of model variables (*R* ^2^_adj_).

Abbreviation: dbMEM, Moran's Eigenvector Map.

* *p* < 0.05.

*** *p* < 0.001.

## 4 Discussion

### 4.1 Alpha Diversity

Despite the general similarity in agricultural practices of orchards belonging to the same regional growers' association, orchards showed a huge idiosyncrasy as indicated by the variability in alpha diversity, with single orchards having only a small part of the total diversity. Similarly, environmental variables showed a large variability, potentially leading to the observed differences. The ranking of orchards with alpha diversity was different between prokaryotes, protists and fungi, indicating that organisms responded differently to environmental conditions. It is well known that univariate indices typically generate heterogenous results for a range of soil taxa (Gao et al. 2025; Tsiafouli et al. 2015). Fungi are more influenced by agricultural management of apple orchards than bacteria (Hulsmans et al. 2022) and a meta‐analysis found that agricultural practices affect soil organisms differently, with studies showing contrasting results, indicative of site‐ and cultivation‐specific effects (Lori et al. 2025). Therefore, our first hypothesis (H1) was supported: prokaryotes, protists and fungi responded differently to abiotic soil characteristics despite uniform agricultural management.

As we should resist the oversimplification that a high‐diversity microbial community is ‘better’ than a low‐diversity community (Shade 2017), especially orchard ID1 had the highest richness of unique prokaryotic ASVs, lowest fungal alpha diversity and lowest richness of unique fungal ASVs. Orchard ID1 also showed the lowest P/A ratio and the most extreme values in environmental variables like orchards ID23 and ID24. These results indicated deficiencies in soil properties of orchard ID1.

### 4.2 Community Composition

Few orchard‐specific ASVs accounted for a substantial portion of total abundance. The most abundant unique ASVs belonged to phyla well‐documented in soil, such as Chloroflexi and Firmicutes (Liu et al. 2024), Rhodophyta (Joseph and Ray 2024) and Chytridiomycota (Qin et al. 2016), making their occurrence in these orchards expected.

Among prokaryotes, Acidobacteriota, Actinobacteriota and Proteobacteria were most abundant in all sites. Similar dominance was found in apple orchards from Poland (Bokszczanin et al. 2023) and Northern Italy (Peruzzi et al. 2017). Actinobacteriota are prominent degraders of plant residues (Lewin et al. 2016), Proteobacteria and Acidobacteriota are involved in biogeochemical cycling (Spain et al. 2009) and Proteobacteria are among the phyla with the highest correlation to metals in soils (Liu et al. 2021). These properties can explain their general dominance in studied orchards. Furthermore, the relatively low abundance of Crenarchaeota was consistent with values reported for global archaeal soil populations (Bates et al. 2011). Despite their low abundance in soils, Crenarchaeota play a major role in biogeochemical cycling (Offre et al. 2013). Some of the core bacterial ASVs of our study were among the most abundant genera in North‐Italian (*Gaiella*, *Microluntus*, *Solirubrobacter*, *Pirellula*; Peruzzi et al. 2017) and Canadian apple orchards (*Gaiella*; Ajeethan et al. 2023) and occur globally (Delgado‐Baquerizo et al. 2020), indicating their widespread occurrence. Taxa of genus *Gaiella* absorb phosphate (Severino et al. 2019) and produce antibiotics to inhibit the growth of other microorganisms such as fungi (Ouchene et al. 2022; Zhao et al. 2019). *Pedomicrobium* is involved in the precipitation or oxidation of Fe and/or Mn (Cox and Sly 1997). *Candidus Xiphinematobacter* is a putative endosymbiont bacterium associated with soil nematodes belonging to the *Xiphinema‐americanum* group (Lazarova et al. 2016). These nematodes, which occur in Italy, are migratory ectoparasites of plant roots (EFSA 2018). Although our study focused on prokaryotes, protists and fungi, detecting sequence signatures of this endosymbiont points to the presence of these ectoparasitic nematodes. Including additional organismal groups with different body sizes, such as soil nematodes, would therefore represent a valuable next step to validate orchard differences across broader taxonomic and functional size spectrums. These core prokaryotic ASVs indicate the intricate relationships between prokaryotes and other soil organisms and their high adaptation to environmental conditions.

Protists represent an often‐overlooked component of the soil microbiome when trying to improve crop yields through approaches such as biofertilization or biocontrol (Chandarana and Amaresan 2022). At a global scale across different climates, Amoebozoa, Cercozoa, Ciliophora, Apicomplexa are among the most important soil protists (Oliverio et al. 2020). These groups were prominent in all studied orchards. Most protists are consumers, although parasites and phototrophs occur as well, but mostly in tropical and arid ecosystems (Oliverio et al. 2020). Here, five (ID 7, 9, 10, 13, 14) of the eight orchards with a dominance of phototrophic Chlorophyta were positively related to high pH, clay and conductivity. The abundance of soil phototrophs increases with pH (Sauze et al. 2017), clay plays an important role in the release and availability of soil nutrients (Kome et al. 2019) and conductivity is related to clay (Heil and Schmidhalter 2017). The observed dominance of Chlorophyta in these soils may result from the direct effects of nutrient availability or indirect effects, such as biotic interaction with bacteria. Among genera of the core protist ASVs, one taxon (*Pythium*, a fungus‐like protist) causes root rot of young apple trees (Utkhede and Smith 1991), three are bacteria‐eating amoebozoa (*Nematostelium*, *Filamoeba*, *Ceratiomyxcella*) and two are potentially mixotrophic soil algae (*Vischeria*, *Chlorosarcinopsis*; Cherdchukeattisak et al. 2018). Protists are important for plant disease control and plant growth promotion, possibly by feeding on plant pathogenic bacteria or by stimulating plant growth‐promoting bacteria (Sun et al. 2021). These core protist taxa were representative of the different aspects of protist diversity comprising positive and negative interactions with soil organisms and plants.

Fungi interact with and influence the activity and functions of microbial prokaryotes and eukaryotes in most ecosystems (Bahram and Netherway 2022). In line with observations from other aged apple orchards (Franke‐Whittle et al. 2015; Xu et al. 2023), Ascomycota was the dominant fungal phylum in our study. Members of this phylum contribute significantly to soil fertility by degrading organic matter within the rhizosphere (Schoch et al. 2009). However, agricultural soils also show abundant plant pathogens (Lal 2015). Rozellomycota, which are obligate pathogens on eukaryotes such as amoebae, algae and fungi and preferentially consume organic debris (Tedersoo et al. 2017), were abundant in two orchards (ID1, ID2). These results further indicated that orchard ID1 exhibited a disrupted microbial community. Furthermore, most core fungi were plant pathogens (e.g., *Alternaria*, *Cladosporium* or *Fusarium*). While apple replant disease (ARD) is widely recognized as a multi‐causal disease complex caused by interactions among bacteria, oomycetes, nematodes and fungi (Mazzola and Manici 2012; Winkelmann et al. 2019), the prevalence of these specific fungal taxa highlights their potential contribution to ARD symptoms (Xiang et al. 2021) in the studied soils. This high prevalence of plant pathogens in soils likely resulted from multi‐year apple cultivation, as found in Canadian apple orchards (Ali et al. 2025).

In functional prediction using FunGuild most reads remained unassigned, a typical issue with environmental samples such as soil (Nguyen et al. 2016; Tedersoo et al. 2014). The dominance of saprotrophic and pathotrophic guilds aligned with other studies on orchard soils (Xu et al. 2023; Zarrabian and Sherif 2026). The low abundance of mycorrhizal fungi (< 1%) across orchards may be attributed to intensive management practices such as chemical‐only fertilization, which is known to suppress mycorrhizal associations (Jiang et al. 2021). As AMF are highly responsive to site and management characteristics of apple orchards (Turrini et al. 2017), the complete absence of mycorrhizal fungi in orchard ID1 provided further evidence of microbial dysbiosis in this specific orchard.

### 4.3 Influence of Environmental Factors on Alpha‐Diversity and Community Composition

Soil texture (clay), altitude as proxy for air temperature, heavy metals, conductivity, pH and nutrients were related to alpha‐diversity and community composition of prokaryotes, protists and fungi. Even though these factors are all important for soil diversity (Delgado‐Baquerizo et al. 2018), not all variables were equally important for organisms and showed opposite relationships. Richness of fungi increased with increasing altitude while that of prokaryotes decreased and Cu was linked to communities of prokaryotes and fungi while organic matter, conductivity and orchards' age were only important for fungi. These results further supported H1. Fungi (Cordero et al. 2023) are favoured by colder temperatures while bacteria (Metze et al. 2024) by warmer ones. Bacteria are better at decomposing simple organic substrates, while fungi at decomposing complex organic substrates (Wang and Kuzyakov 2024). Heavy metals such as Cu and Zn originating from pesticides applied in vineyards and orchards (Fishel 2014) have multiple effects on soil microorganisms including a decrease in diversity and abundance, an increase in metal resistant bacteria, a change in metabolic activities (Sazykin et al. 2023), a decrease in microbial diversity and detritus processing (Vázquez‐Blanco et al. 2020) and a reduction of invertebrate bioturbation activities (Hunting et al. 2013). The long‐term cultivation of one crop can lead to the accumulation of pathogenic fungi (Pervaiz et al. 2020). These relationships can be linked to our results. Thus, our study showed that a factor that seems unimportant for a certain group is important for another one and only a study covering different soil organisms can assess the different aspects of soil characteristics on microbial diversity.

### 4.4 Beta‐Diversity

Protists showed the lowest mean beta‐diversity and a lower spatial distance decay than prokaryotes, indicating that protists showed a lower spatial turnover than prokaryotes. Sloan's neutral modelling framework indicated that prokaryotes showed the highest immigration rate, followed by protists and fungi. For prokaryotes and protists, this gradient in immigration strength aligned with the strength of distance decay: the lower the immigration rate, the weaker the distance decay. In contrast, fungi did not show any distance decay. In urban parks (spatial distance < 25 km), spatial distance decay was likewise stronger for bacteria than for protists, while fungi did not show any distance decay (Isabwe et al. 2022). In near‐urban farmlands over larger spatial scales (> 1600 km; Ren et al. 2023), generalist fungi showed a faster decay than generalist bacteria. We suggest that the extent of spatial scale and habitat type determine these differences between studies. However, in vineyards (Larsen et al. 2023), both soil fungi and soil bacteria exhibited higher migration rates than in our study and fungi dispersed better than bacteria, contrary to our findings. Spatial scale, organism properties, study region and ecosystem determine the distance decay relationship (Soininen et al. 2007). Thus, this discrepancy might be an effect of spatial scale (Larsen et al. 2023: maximum distance ~15 km; this study: maximum distance ~65 km) and different cultivation systems (grape: Larsen et al. 2023; apple: this study).

The niche breadth of generalist prokaryotes and protists was larger than that of generalist fungi, implying stronger adaptation of the former two than the latter. A similar result was found for soils of non‐forested sites along an altitudinal gradient in the Swiss Alps (Malard et al. 2022) and can be related to bacteria‐protist interactions (Malard et al. 2022) and to the general high adaptation potential of bacteria (Moreno‐Gámez 2022).

With both incidence and abundance data, replacement of taxa (i.e., turnover/balanced variation in species abundance) dominated for prokaryotes, protists and fungi. This pattern indicated that communities did not represent nested subsets of a common pool but were shaped by strong species turnover between orchards. In fact, species turnover is an organismal group‐overarching mechanism shaping beta‐diversity (Soininen et al. 2018).

As revealed by variance partitioning, spatial variables contributed to community composition and abundance only in prokaryotes and protists, though their importance was minor compared to environmental factors. Remarkably, large‐scale spatial patterns were mainly important for prokaryotes whereas small‐scale spatial patterns prevailed for protists. We suggest that these differences relate to the better dispersal of prokaryotes than protists in soil. Similar findings have been reported for marine bacteria and protists (Wu et al. 2018).

It is largely accepted that soil communities are assembled by both stochastic and deterministic processes (Yang et al. 2022). Here, soil organisms were mainly assembled by stochastic processes, even though deterministic processes were also important. Similar results were found for bacteria and fungi in vineyards (Larsen et al. 2023), bacteria and fungi in apple orchards (Zheng et al. 2021) and protists in forest soils (Bahram et al. 2016), but not for bacteria and fungi in near‐urban farmlands (Ren et al. 2023). While the relative dominance of stochastic versus deterministic processes in community assembly remains a subject of debate (Menéndez‐Serra et al. 2023), growing evidence suggests that stochasticity prevails in apple orchards (Zheng et al. 2021; this study). The predominance of stochastic assembly in prokaryotes, protists and fungi supported the size‐plasticity hypothesis, which suggests that microorganisms are mainly dispersal‐driven rather than environmentally filtered, owing to their high metabolic flexibility and environmental tolerance compared to macroscopic organisms (Farjalla et al. 2012).

Regarding our second hypothesis (H2), site‐specific physico‐chemical soil parameters explained a larger proportion of community variation than geographic distance for prokaryotes and protists. However, fungi represented a clear exception: they exhibited no spatial distance decay and geographic space accounted for virtually no variation in their community structure. This indicates that fungal dispersal is not distance‐constrained at the studied spatial scale (~65 km), causing their assembly to be uncoupled from geographic distance. Furthermore, the low number of core prokaryotic, protist and fungal ASVs in apple orchards likely stems from stochastic community assembly coupled with generally low dispersal rates.

### 4.5 Putative Functions of Prokaryotes

Aerobic and anaerobic chemoheterotrophy were well represented (~13%–15%) in the studied orchards, which aligns with other studies (Wu et al. 2026; Zhang et al. 2022) reporting chemoheterotrophic metabolism to dominate bacterial functional profiles in managed soils. Furthermore, the substantial proportion of nitrification and aerobic ammonia oxidation (15% ± 4.6%) indicated a dynamic nitrogen‐cycling community, as frequently documented in fertilized orchard systems (Wu et al. 2026; Wang et al. 2024). Conversely, specialized functions such as dark hydrogen oxidation and animal parasites/symbionts maintained lower baseline abundances (< 5%), consistent with baseline functional levels reported for healthy agroecosystems and orchard soils (Sansupa et al. 2021; Wang et al. 2024). Here, only in orchard ID23, dark hydrogen oxidation was a major function. Hydrogen is produced by nitrogen fixation, fermentation reactions or deposition from the atmosphere and can be taken up by hydrogen oxidizing bacteria (dark hydrogen oxidation) that may also act as plant‐growth promoters (Islam et al. 2023). This aspect may require more attention. Furthermore, orchard ID1 stood out having the lowest (aerobic ammonia oxidation, nitrification) and highest (anaerobic chemoheterotrophy) levels of certain functions, pointing to an unbalanced microbial community. These local functional differences in orchards ID1 and ID23 further supported H1 as site‐specific abiotic soil conditions shaped bacterial functional profiles despite overall uniform agricultural management practices.

### 4.6 Summary

As hypothesised, prokaryotes, protists and fungi exhibited distinct patterns regarding alpha‐ and beta‐diversity, yet all showed stochastic community assembly alongside low dispersal rates, which likely determined the low richness of core communities. Soil texture, altitude as proxy for temperature and heavy metals were associated with all three organismal groups, whereas other environmental factors were group‐specific. Our findings indicated that strategies aimed at modifying soil microbiomes should account for the idiosyncrasy of local conditions, as prokaryotes, protists and fungi respond differently to environmental drivers.

## Author Contributions

**Ulrike Obertegger:** conceptualization, formal analysis, writing – review and editing. **Emanuela Coller:** conceptualization, formal analysis, writing – review and editing. **Sara Zanoni:** data curation, validation, formal analysis. **Roberto Zanzotti:** data curation, validation, formal analysis. **Raffaella Morelli:** data curation, validation, formal analysis. **Massimo Pindo:** data curation, validation, formal analysis. **Loris Marchel:** conceptualization, writing – review and editing, formal analysis. **Daniele Prodorutti:** conceptualization, formal analysis, writing – review and editing.

## Conflicts of Interest

The authors declare no conflicts of interest.

## Supporting information


**Figure S1:** Sampling sites and their site ID. Numbers go from 1 to 26.
**Figure S2:** Correlation plot of all environmental variables assessed. Shown are only significant correlations (*p* < 0.05); soil organic matter (SOM), total nitrogen (tot.N), active CaCO_3_ (act_CaCO_3_).
**Figure S3:** RDA of (A) prokaryotes, (B) protists, (C) fungi. Colours are sequential (from light to dark) for altitude (A, B) and age (C); conductivity (cond), altitude (alti).
**Table S1:** Details on primers used for high‐throughput sequencing.
**Table S2:** Read number threshold for rarefying to even depth, number of ASVs obtained across all orchards, % taxonomic coverage of reads. *For protists, taxonomic assignment with PR2 leads to the filling of unknown levels with the taxonomic assignment of the previous level and an _X is appended and this propagates down with _XX, _XXX and so forth until a level is again taxonomically assigned. Therefore, we manually curated the protist dataset at genus level.
**Table S3:** Relative abundance of the 10 most abundant prokaryotic phyla for each site.
**Table S4:** Number of prokaryotic ASVs that occurred in all sites and their phylum assignment.
**Table S5:** Taxonomic assignment of the 74 prokaryotic core ASVs occurring in all sites. The last column indicates genera that occur globally in soils (total number of globally occurring dominant genera is 66; Delgado‐Baquerizo et al. 2018).
**Table S6:** Relative abundance of the 10 most abundant protist phyla for each site.
**Table S7:** Relative abundance of protist phyla in each site. Colours indicate the four types of phyla combinations (Group 1: Alveolata, Evosea and Rhizaria [the first 14 sites]; Group 2: Alveolata, Chlorophyta and Evosea [the next 5 sites]; Group 3: Alveolata, Chlorophyta and Rhizaria [the next 3 sites]; Group 4: Chlorophyta, Evosea, Rhizaria [ID5]; group5: Alveolata, Chlorophyta, Evosea [ID11]; Group 6: Alveolata, Evoseas, Stramenopiles [ID22].
**Table S8:** Taxonomic assignment of the nine core protist ASVs occurring in all sites.
**Table S9:** Total number of unique protist ASVs per site, number of unique ASVs per site and their phylum assignment and their % with respect to total number of unique ASVs per site.
**Table S10:** Relative abundance of fungal phyla in each site.
**Table S11:** Taxonomic assignment of the 13 core fungal ASVs occurring in all sites.
**Table S12:** Total number of unique fungal ASVs per site, number of unique ASVs per site and their phylum assignment and their % with respect to total number of unique ASVs per site.
**Table S13:** % contribution of turnover and balanced variation in species abundance, respectively, to beta‐diversity of the four organismal groups.

## Data Availability

The sequence data for this study have been deposited in the European Nucleotide Archive (ENA; https://www.ebi.ac.uk/ena/browser/home) at EMBL‐EBI under accession number PRJEB84039 for 16S, PRJEB84052 for 18S and PRJEB84059 for ITS data.
