# Supplementary material for: Drivers of Soil Community Composition in Apple Orchards: Insights From Environmental Metabarcoding
Source: Environ Microbiol Rep. 2026 Oct 5;18(5):e70422. doi: 10.1111/1758-2229.70422 (PMC13635805; doi:10.1111/1758-2229.70422)
Supplement: Supplementary file 1 [file EMI4-18-e70422-s001.docx]

**Drivers of soil community composition in apple orchards: insights from environmental metabarcoding**

Obertegger Ulrike^1*^, Coller Emanuela^2*^, Zanoni Sara^2^, Zanzotti Roberto^2^, Morelli Raffaella^2^, Pindo Massimo^3^, Marchel Loris^4^, Prodorutti Daniele^2^

^1^ Fondazione Edmund Mach, Research and Innovation Centre, Research Group Hydrobiology, San Michele all’Adige, Italy

^2^ Fondazione Edmund Mach, Technological Transfer Centre, Organic Agriculture and Soil Quality Unit, San Michele all’Adige, Italy

^3^ Fondazione Edmund Mach, Research and Innovation Centre, Computational Biology, San Michele all’Adige, Italy

^4^ APOT, Associazione Produttori Ortofrutticoli Trentini, Trento, Italy

**Supplementary Material**

**Figures**

Supplementary Figure 1. Sampling sites and their site ID. Numbers go from 1 to 26.

Supplementary Figure 2. Correlation plot of all environmental variables assessed. Shown are only significant correlations (P < 0.05); soil organic matter (SOM), total nitrogen (tot.N), active CaCO3 (act_CaCO3).

Supplementary Figure 3. RDA of (A) prokaryotes, (B) protists, (C) fungi. Colours are sequential (from light to dark) for altitude (A, B) and age (C); conductivity (cond), altitude (alti).

**Tables**

Supplementary Table 1. Details on primers used for high-throughput sequencing.

| type | name | sequence | citation |
| --- | --- | --- | --- |
| 16S | 515F | 5′-GTGYCAGCMGCCGCGGTAA-3 | Apprill et al. 2015; Parada et al. 2016 |
|  | 806R | 5′-GGACTACNVGGGTWTCTAAT-3′ |  |
| 18S | V4F | 5′-CCAGCASCYGCGGTAATTCC-3′ | Stoeck et al. 2010 |
|  | V4R | 5′-ACTTTCGTTCTTGATYRATGA-3′ |  |
| ITS | ITS1F | 5′-CTTGGTCATTTAGAGGAAGTAA-3′ | Gardes et al. 1993; White et al. 1990 |
|  | ITS2 | 5′-GCTGCGTTCTTCATCGATGC-3′ |  |

Apprill, A.; Mcnally, S.; Parsons, R.; Weber, L. Minor revision to V4 region SSU rRNA 806R gene primer greatly increases detection of SAR11 bacterioplankton. Aquat. Microb. Ecol. 2015, 75, 129–137.

Gardes, M.; Bruns, T.D. ITS primers with enhanced specificity for basidiomycetes - Application to the identification of mycorrhizae and rusts. Mol. Ecol. 1993, 2, 113–118.

Parada, A.E.; Needham, D.M.; Fuhrman, J.A. Every base matters: Assessing small subunit rRNA primers for marine microbiomes with mock communities, time series and global field samples. Environ. Microbiol. 2016, 18, 1403–1414.

Stoeck, T., Bass, D., Nebel, M., Christen, R., Jones, M. D. M., Breiner, H.-W., and Richards, T. A. 2010. Multiple marker parallel tag environmental DNA sequencing reveals a highly complex eukaryotic community in marine anoxic water., Molecular Ecology, 19 Suppl 1, 21–31, https://doi.org/10.1111/j.1365-294X.2009.04480.x, http://www.ncbi.nlm.nih.gov/pubmed/20331767,

White, T.J.; Bruns, T.; Lee, S.; Taylor, J. Amplification and Direct Sequencing of Fungal	Ribosomal RNA Genes for Phylogenetics. In PCR Protocols: A Guide to Methods Applications; Innis, M.A., Gelfand, D.H., Snisky, J.J., White, T.J., Eds.; Academic Press: New Yourk, NY, USA, 1990; pp. 315–322.

Supplementary Table 2. Read number threshold for rarefying to even depth, number of ASVs obtained across all orchards, % taxonomic coverage of reads. *For protists, taxonomic assignment with PR2 leads to the filling of unknown levels with the taxonomic assignment of the previous level and an _X is appended and this propagates down with _XX, _XXX etc. until a level is again taxonomically assigned. Therefore, we manually curated the protist dataset at genus level.

|  | | prokaryotes | protists | fungi |
| --- | --- | --- | --- | --- |
| read number threshold for rarefying to even depth | | 41,351 | 15,058 | 42,579 |
| total number of ASVs | | 11980 | 6538 | 6538 |
| assigned at the level of | phylum (%) | 91 | 100 | 85 |
|  | class (%) | 86 | * | 79 |
|  | order (%) | 73 | * | 77 |
|  | family (%) | 56 | * | 74 |
|  | genus (%) | 23 | 79 | 70 |

Supplementary Table 3. Relative abundance of the ten most abundant prokaryotic phyla for each site.

| site ID | Phylum | Phylum relative abundance | rank |
| --- | --- | --- | --- |
| 1 | Acidobacteriota | 29.7 | 10 |
| 1 | Actinobacteriota | 10.3 | 8 |
| 1 | Chloroflexi | 10.0 | 7 |
| 1 | Crenarchaeota | 3.4 | 5 |
| 1 | Firmicutes | 1.9 | 2 |
| 1 | Methylomirabilota | 3.0 | 3 |
| 1 | Myxococcota | 1.4 | 1 |
| 1 | Planctomycetota | 6.8 | 6 |
| 1 | Proteobacteria | 19.8 | 9 |
| 1 | Verrucomicrobiota | 3.1 | 4 |
| 2 | Acidobacteriota | 16.7 | 8 |
| 2 | Actinobacteriota | 21.5 | 10 |
| 2 | Chloroflexi | 8.3 | 6 |
| 2 | Crenarchaeota | 17.9 | 9 |
| 2 | Firmicutes | 4.0 | 4 |
| 2 | Methylomirabilota | 1.8 | 1 |
| 2 | Myxococcota | 2.2 | 3 |
| 2 | Planctomycetota | 5.7 | 5 |
| 2 | Proteobacteria | 14.9 | 7 |
| 2 | Verrucomicrobiota | 2.0 | 2 |
| 3 | Acidobacteriota | 22.4 | 10 |
| 3 | Actinobacteriota | 16.1 | 8 |
| 3 | Chloroflexi | 9.2 | 6 |
| 3 | Crenarchaeota | 9.9 | 7 |
| 3 | Firmicutes | 1.9 | 2 |
| 3 | Methylomirabilota | 3.8 | 4 |
| 3 | Myxococcota | 1.8 | 1 |
| 3 | Planctomycetota | 5.9 | 5 |
| 3 | Proteobacteria | 18.6 | 9 |
| 3 | Verrucomicrobiota | 3.4 | 3 |
| 4 | Acidobacteriota | 16.9 | 9 |
| 4 | Actinobacteriota | 22.8 | 10 |
| 4 | Chloroflexi | 10.4 | 6 |
| 4 | Crenarchaeota | 15.6 | 7 |
| 4 | Firmicutes | 1.7 | 2 |
| 4 | Methylomirabilota | 4.0 | 5 |
| 4 | Myxococcota | 1.5 | 1 |
| 4 | Planctomycetota | 3.5 | 4 |
| 4 | Proteobacteria | 16.4 | 8 |
| 4 | Verrucomicrobiota | 2.3 | 3 |
| 5 | Acidobacteriota | 20.3 | 10 |
| 5 | Actinobacteriota | 13.9 | 8 |
| 5 | Chloroflexi | 10.3 | 6 |
| 5 | Crenarchaeota | 10.9 | 7 |
| 5 | Firmicutes | 2.2 | 2 |
| 5 | Methylomirabilota | 3.0 | 3 |
| 5 | Myxococcota | 2.0 | 1 |
| 5 | Planctomycetota | 7.5 | 5 |
| 5 | Proteobacteria | 16.8 | 9 |
| 5 | Verrucomicrobiota | 3.8 | 4 |
| 6 | Acidobacteriota | 15.4 | 8 |
| 6 | Actinobacteriota | 16.2 | 9 |
| 6 | Chloroflexi | 6.5 | 4 |
| 6 | Crenarchaeota | 14.6 | 7 |
| 6 | Firmicutes | 2.8 | 3 |
| 6 | Methylomirabilota | 2.0 | 1 |
| 6 | Myxococcota | 2.1 | 2 |
| 6 | Planctomycetota | 7.7 | 5 |
| 6 | Proteobacteria | 18.5 | 10 |
| 6 | Verrucomicrobiota | 10.1 | 6 |
| 7 | Acidobacteriota | 16.0 | 8 |
| 7 | Actinobacteriota | 22.0 | 9 |
| 7 | Chloroflexi | 7.1 | 6 |
| 7 | Crenarchaeota | 4.9 | 4 |
| 7 | Firmicutes | 1.7 | 1 |
| 7 | Methylomirabilota | 2.5 | 2 |
| 7 | Myxococcota | 2.8 | 3 |
| 7 | Planctomycetota | 6.9 | 5 |
| 7 | Proteobacteria | 22.6 | 10 |
| 7 | Verrucomicrobiota | 8.8 | 7 |
| 8 | Acidobacteriota | 14.6 | 7 |
| 8 | Actinobacteriota | 26.8 | 10 |
| 8 | Chloroflexi | 7.1 | 6 |
| 8 | Crenarchaeota | 18.1 | 9 |
| 8 | Firmicutes | 1.2 | 2 |
| 8 | Methylomirabilota | 1.8 | 3 |
| 8 | Myxococcota | 1.1 | 1 |
| 8 | Planctomycetota | 3.5 | 4 |
| 8 | Proteobacteria | 16.8 | 8 |
| 8 | Verrucomicrobiota | 5.0 | 5 |
| 9 | Acidobacteriota | 15.8 | 8 |
| 9 | Actinobacteriota | 26.7 | 10 |
| 9 | Chloroflexi | 6.6 | 6 |
| 9 | Crenarchaeota | 21.0 | 9 |
| 9 | Firmicutes | 1.9 | 3 |
| 9 | Methylomirabilota | 1.4 | 1 |
| 9 | Myxococcota | 1.4 | 2 |
| 9 | Planctomycetota | 4.5 | 5 |
| 9 | Proteobacteria | 13.8 | 7 |
| 9 | Verrucomicrobiota | 3.8 | 4 |
| 10 | Acidobacteriota | 22.3 | 10 |
| 10 | Actinobacteriota | 17.4 | 8 |
| 10 | Chloroflexi | 10.0 | 7 |
| 10 | Crenarchaeota | 4.4 | 4 |
| 10 | Firmicutes | 1.3 | 1 |
| 10 | Methylomirabilota | 2.4 | 3 |
| 10 | Myxococcota | 2.3 | 2 |
| 10 | Planctomycetota | 9.0 | 6 |
| 10 | Proteobacteria | 20.4 | 9 |
| 10 | Verrucomicrobiota | 5.2 | 5 |
| 11 | Acidobacteriota | 14.1 | 7 |
| 11 | Actinobacteriota | 24.3 | 10 |
| 11 | Chloroflexi | 6.4 | 6 |
| 11 | Crenarchaeota | 15.9 | 8 |
| 11 | Firmicutes | 2.6 | 2 |
| 11 | Methylomirabilota | 0.5 | 1 |
| 11 | Myxococcota | 3.5 | 4 |
| 11 | Planctomycetota | 6.4 | 5 |
| 11 | Proteobacteria | 18.5 | 9 |
| 11 | Verrucomicrobiota | 3.0 | 3 |
| 12 | Acidobacteriota | 14.7 | 8 |
| 12 | Actinobacteriota | 27.2 | 10 |
| 12 | Chloroflexi | 7.6 | 6 |
| 12 | Crenarchaeota | 21.8 | 9 |
| 12 | Firmicutes | 2.4 | 3 |
| 12 | Methylomirabilota | 1.9 | 2 |
| 12 | Myxococcota | 1.2 | 1 |
| 12 | Planctomycetota | 2.8 | 5 |
| 12 | Proteobacteria | 14.6 | 7 |
| 12 | Verrucomicrobiota | 2.8 | 4 |
| 13 | Acidobacteriota | 17.3 | 8 |
| 13 | Actinobacteriota | 26.2 | 10 |
| 13 | Chloroflexi | 8.1 | 6 |
| 13 | Crenarchaeota | 11.2 | 7 |
| 13 | Firmicutes | 1.3 | 1 |
| 13 | Methylomirabilota | 1.4 | 2 |
| 13 | Myxococcota | 3.3 | 4 |
| 13 | Planctomycetota | 6.0 | 5 |
| 13 | Proteobacteria | 18.3 | 9 |
| 13 | Verrucomicrobiota | 2.6 | 3 |
| 14 | Acidobacteriota | 15.9 | 8 |
| 14 | Actinobacteriota | 24.5 | 10 |
| 14 | Chloroflexi | 8.7 | 6 |
| 14 | Crenarchaeota | 10.6 | 7 |
| 14 | Firmicutes | 1.4 | 1 |
| 14 | Methylomirabilota | 2.1 | 2 |
| 14 | Myxococcota | 2.7 | 3 |
| 14 | Planctomycetota | 6.2 | 5 |
| 14 | Proteobacteria | 18.8 | 9 |
| 14 | Verrucomicrobiota | 3.9 | 4 |
| 15 | Acidobacteriota | 15.2 | 7 |
| 15 | Actinobacteriota | 24.8 | 10 |
| 15 | Chloroflexi | 7.0 | 6 |
| 15 | Crenarchaeota | 18.0 | 9 |
| 15 | Firmicutes | 2.0 | 3 |
| 15 | Methylomirabilota | 1.7 | 2 |
| 15 | Myxococcota | 1.4 | 1 |
| 15 | Planctomycetota | 4.4 | 4 |
| 15 | Proteobacteria | 15.4 | 8 |
| 15 | Verrucomicrobiota | 6.5 | 5 |
| 16 | Acidobacteriota | 15.6 | 8 |
| 16 | Actinobacteriota | 21.8 | 10 |
| 16 | Chloroflexi | 6.8 | 5 |
| 16 | Crenarchaeota | 15.1 | 7 |
| 16 | Firmicutes | 3.5 | 3 |
| 16 | Methylomirabilota | 1.4 | 1 |
| 16 | Myxococcota | 2.5 | 2 |
| 16 | Planctomycetota | 4.8 | 4 |
| 16 | Proteobacteria | 17.3 | 9 |
| 16 | Verrucomicrobiota | 7.1 | 6 |
| 17 | Acidobacteriota | 17.8 | 9 |
| 17 | Actinobacteriota | 20.6 | 10 |
| 17 | Chloroflexi | 7.6 | 6 |
| 17 | Crenarchaeota | 15.1 | 7 |
| 17 | Firmicutes | 1.1 | 1 |
| 17 | Methylomirabilota | 2.1 | 2 |
| 17 | Myxococcota | 2.3 | 3 |
| 17 | Planctomycetota | 6.3 | 4 |
| 17 | Proteobacteria | 16.0 | 8 |
| 17 | Verrucomicrobiota | 7.2 | 5 |
| 18 | Acidobacteriota | 15.1 | 7 |
| 18 | Actinobacteriota | 24.9 | 10 |
| 18 | Chloroflexi | 7.3 | 6 |
| 18 | Crenarchaeota | 15.5 | 8 |
| 18 | Firmicutes | 3.6 | 3 |
| 18 | Methylomirabilota | 1.3 | 1 |
| 18 | Myxococcota | 2.0 | 2 |
| 18 | Planctomycetota | 4.2 | 4 |
| 18 | Proteobacteria | 17.3 | 9 |
| 18 | Verrucomicrobiota | 5.0 | 5 |
| 19 | Acidobacteriota | 17.0 | 8 |
| 19 | Actinobacteriota | 21.4 | 10 |
| 19 | Chloroflexi | 7.7 | 6 |
| 19 | Crenarchaeota | 20.8 | 9 |
| 19 | Firmicutes | 2.7 | 3 |
| 19 | Methylomirabilota | 1.5 | 1 |
| 19 | Myxococcota | 1.6 | 2 |
| 19 | Planctomycetota | 3.2 | 4 |
| 19 | Proteobacteria | 14.1 | 7 |
| 19 | Verrucomicrobiota | 6.0 | 5 |
| 20 | Acidobacteriota | 16.6 | 9 |
| 20 | Actinobacteriota | 27.0 | 10 |
| 20 | Chloroflexi | 8.2 | 6 |
| 20 | Crenarchaeota | 15.9 | 8 |
| 20 | Firmicutes | 1.5 | 1 |
| 20 | Methylomirabilota | 1.9 | 3 |
| 20 | Myxococcota | 1.6 | 2 |
| 20 | Planctomycetota | 4.9 | 5 |
| 20 | Proteobacteria | 14.6 | 7 |
| 20 | Verrucomicrobiota | 3.9 | 4 |
| 21 | Acidobacteriota | 16.2 | 8 |
| 21 | Actinobacteriota | 18.0 | 9 |
| 21 | Chloroflexi | 6.9 | 5 |
| 21 | Crenarchaeota | 15.3 | 7 |
| 21 | Firmicutes | 2.7 | 3 |
| 21 | Methylomirabilota | 2.2 | 2 |
| 21 | Myxococcota | 1.7 | 1 |
| 21 | Planctomycetota | 5.6 | 4 |
| 21 | Proteobacteria | 19.1 | 10 |
| 21 | Verrucomicrobiota | 7.8 | 6 |
| 22 | Acidobacteriota | 14.9 | 8 |
| 22 | Actinobacteriota | 18.1 | 9 |
| 22 | Chloroflexi | 5.7 | 4 |
| 22 | Crenarchaeota | 14.4 | 7 |
| 22 | Firmicutes | 4.8 | 3 |
| 22 | Methylomirabilota | 1.6 | 1 |
| 22 | Myxococcota | 2.3 | 2 |
| 22 | Planctomycetota | 6.0 | 5 |
| 22 | Proteobacteria | 20.8 | 10 |
| 22 | Verrucomicrobiota | 6.8 | 6 |
| 23 | Acidobacteriota | 15.2 | 8 |
| 23 | Actinobacteriota | 27.6 | 10 |
| 23 | Chloroflexi | 7.5 | 6 |
| 23 | Crenarchaeota | 12.7 | 7 |
| 23 | Firmicutes | 2.6 | 3 |
| 23 | Methylomirabilota | 2.5 | 1 |
| 23 | Myxococcota | 2.6 | 2 |
| 23 | Planctomycetota | 3.4 | 4 |
| 23 | Proteobacteria | 17.5 | 9 |
| 23 | Verrucomicrobiota | 4.9 | 5 |
| 24 | Acidobacteriota | 15.7 | 7 |
| 24 | Actinobacteriota | 22.3 | 10 |
| 24 | Chloroflexi | 7.8 | 6 |
| 24 | Crenarchaeota | 16.0 | 8 |
| 24 | Firmicutes | 3.7 | 3 |
| 24 | Methylomirabilota | 1.8 | 1 |
| 24 | Myxococcota | 1.8 | 2 |
| 24 | Planctomycetota | 4.4 | 4 |
| 24 | Proteobacteria | 17.4 | 9 |
| 24 | Verrucomicrobiota | 5.1 | 5 |
| 25 | Acidobacteriota | 14.4 | 7 |
| 25 | Actinobacteriota | 18.6 | 9 |
| 25 | Chloroflexi | 6.1 | 6 |
| 25 | Crenarchaeota | 19.7 | 10 |
| 25 | Firmicutes | 5.9 | 5 |
| 25 | Methylomirabilota | 1.8 | 1 |
| 25 | Myxococcota | 2.6 | 2 |
| 25 | Planctomycetota | 4.8 | 3 |
| 25 | Proteobacteria | 17.0 | 8 |
| 25 | Verrucomicrobiota | 5.2 | 4 |
| 26 | Acidobacteriota | 16.0 | 8 |
| 26 | Actinobacteriota | 21.5 | 10 |
| 26 | Chloroflexi | 6.7 | 6 |
| 26 | Crenarchaeota | 12.5 | 7 |
| 26 | Firmicutes | 4.4 | 4 |
| 26 | Methylomirabilota | 2.1 | 2 |
| 26 | Myxococcota | 2.1 | 1 |
| 26 | Planctomycetota | 4.0 | 3 |
| 26 | Proteobacteria | 21.5 | 9 |
| 26 | Verrucomicrobiota | 5.1 | 5 |

Supplementary Table 4. Number of prokaryotic ASVs that occurred in all sites and their phylum assignment.

| Kingdom | phylum | number of ASVs occurring in all sites |
| --- | --- | --- |
| prokaryotes | Acidobacteriota | 22 |
|  | Proteobacteria | 21 |
|  | Actinobacteriota | 14 |
|  | Crenarchaeota | 5 |
|  | Chloroflexi | 2 |
|  | Firmicutes | 2 |
|  | Gemmatimonadota | 2 |
|  | Methylomirabilota | 2 |
|  | Myxococcota | 2 |
|  | Verrucomicrobiota | 2 |
|  | NA | 2 |
|  | Nitrospirota | 1 |
|  | Bacteroidota | 1 |

Supplementary Table 5. Taxonomic assignment of the 74 prokaryotic core ASVs occurring in all sites. The last column indicates genera that occur globally in soils (total number of globally occurring dominant genera is 66; Delgado-Baquerizo *et al.* 2018).

| Phylum | Class | Order | Family | Genus | Delgado-Baquerizo *et al.* 2018 |
| --- | --- | --- | --- | --- | --- |
| Crenarchaeota | Nitrososphaeria | Nitrososphaerales | Nitrososphaeraceae | NA |  |
| Crenarchaeota | Nitrososphaeria | Nitrososphaerales | Nitrososphaeraceae | NA |  |
| Proteobacteria | Alphaproteobacteria | Rhizobiales | NA | NA |  |
| Proteobacteria | Alphaproteobacteria | Rhizobiales | Methyloligellaceae | NA |  |
| Crenarchaeota | Nitrososphaeria | Nitrososphaerales | Nitrososphaeraceae | NA |  |
| Crenarchaeota | Nitrososphaeria | Nitrososphaerales | Nitrososphaeraceae | NA |  |
| Acidobacteriota | Vicinamibacteria | Vicinamibacterales | NA | NA |  |
| Verrucomicrobiota | Verrucomicrobiae | Chthoniobacterales | Xiphinematobacteraceae | Candidatus_Xiphinematobacter |  |
| Actinobacteriota | Actinobacteria | Micrococcales | Micrococcaceae | Pseudarthrobacter |  |
| Proteobacteria | Alphaproteobacteria | Rhizobiales | Xanthobacteraceae | Bradyrhizobium | x |
| Verrucomicrobiota | Verrucomicrobiae | Chthoniobacterales | Xiphinematobacteraceae | Candidatus_Xiphinematobacter |  |
| Actinobacteriota | Thermoleophilia | Gaiellales | Gaiellaceae | Gaiella |  |
| Proteobacteria | Alphaproteobacteria | Rhizobiales | Beijerinckiaceae | Microvirga |  |
| Chloroflexi | KD4-96 | NA | NA | NA |  |
| Actinobacteriota | Thermoleophilia | Gaiellales | Gaiellaceae | Gaiella |  |
| Acidobacteriota | Vicinamibacteria | Vicinamibacterales | NA | NA |  |
| Crenarchaeota | Nitrososphaeria | Nitrososphaerales | Nitrososphaeraceae | NA |  |
| Proteobacteria | Gammaproteobacteria | Steroidobacterales | Steroidobacteraceae | Steroidobacter | x |
| Acidobacteriota | Vicinamibacteria | Vicinamibacterales | Vicinamibacteraceae | NA |  |
| Acidobacteriota | Vicinamibacteria | Vicinamibacterales | NA | NA |  |
| Actinobacteriota | Thermoleophilia | Gaiellales | Gaiellaceae | Gaiella |  |
| Proteobacteria | Alphaproteobacteria | Rhizobiales | Hyphomicrobiaceae | Pedomicrobium | x |
| Acidobacteriota | Vicinamibacteria | Vicinamibacterales | NA | NA |  |
| Proteobacteria | Gammaproteobacteria | Steroidobacterales | Steroidobacteraceae | NA |  |
| Actinobacteriota | NA | NA | NA | NA |  |
| Acidobacteriota | Vicinamibacteria | Vicinamibacterales | Vicinamibacteraceae | NA |  |
| Methylomirabilota | Methylomirabilia | Rokubacteriales | WX65 | NA |  |
| Acidobacteriota | Vicinamibacteria | Subgroup_17 | NA | NA |  |
| Proteobacteria | Alphaproteobacteria | Rhizobiales | Hyphomicrobiaceae | Pedomicrobium |  |
| Nitrospirota | Nitrospiria | Nitrospirales | Nitrospiraceae | Nitrospira |  |
| Gemmatimonadota | Gemmatimonadetes | Gemmatimonadales | Gemmatimonadaceae | NA |  |
| Proteobacteria | Alphaproteobacteria | Tistrellales | Geminicoccaceae | Candidatus_Alysiosphaera |  |
| Acidobacteriota | Vicinamibacteria | Vicinamibacterales | NA | NA |  |
| Actinobacteriota | Thermoleophilia | Gaiellales | Gaiellaceae | Gaiella |  |
| Acidobacteriota | Vicinamibacteria | Vicinamibacterales | NA | NA |  |
| Acidobacteriota | Vicinamibacteria | Vicinamibacterales | NA | NA |  |
| Firmicutes | Bacilli | NA | NA | NA |  |
| Actinobacteriota | Actinobacteria | Pseudonocardiales | Pseudonocardiaceae | NA |  |
| Acidobacteriota | Subgroup_11 | NA | NA | NA |  |
| Acidobacteriota | Vicinamibacteria | Vicinamibacterales | NA | NA |  |
| Acidobacteriota | Vicinamibacteria | Vicinamibacterales | Vicinamibacteraceae | NA |  |
| Acidobacteriota | Vicinamibacteria | Vicinamibacterales | Vicinamibacteraceae | NA |  |
| Acidobacteriota | Vicinamibacteria | Vicinamibacterales | NA | NA |  |
| Firmicutes | Bacilli | Bacillales | Planococcaceae | NA |  |
| Proteobacteria | Alphaproteobacteria | Azospirillales | Azospirillaceae | Skermanella | x |
| Methylomirabilota | Methylomirabilia | Rokubacteriales | NA | NA |  |
| Acidobacteriota | Vicinamibacteria | Subgroup_17 | NA | NA |  |
| Proteobacteria | Gammaproteobacteria | CCD24 | NA | NA |  |
| Proteobacteria | Gammaproteobacteria | Steroidobacterales | Steroidobacteraceae | Steroidobacter | x |
| Actinobacteriota | Actinobacteria | Propionibacteriales | Nocardioidaceae | Nocardioides | x |
| Acidobacteriota | Vicinamibacteria | Vicinamibacterales | Vicinamibacteraceae | NA |  |
| Proteobacteria | Alphaproteobacteria | Rhizobiales | Xanthobacteraceae | NA |  |
| Bacteroidota | Bacteroidia | Cytophagales | Microscillaceae | NA |  |
| Proteobacteria | Alphaproteobacteria | Reyranellales | Reyranellaceae | Reyranella |  |
| Acidobacteriota | Vicinamibacteria | Vicinamibacterales | Vicinamibacteraceae | NA |  |
| Proteobacteria | Gammaproteobacteria | Gammaproteobacteria_Incertae_Sedis | Unknown_Family | Acidibacter |  |
| Actinobacteriota | Acidimicrobiia | Microtrichales | NA | NA |  |
| Acidobacteriota | Vicinamibacteria | Vicinamibacterales | NA | NA |  |
| Proteobacteria | Alphaproteobacteria | Dongiales | Dongiaceae | Dongia |  |
| Proteobacteria | Gammaproteobacteria | Gammaproteobacteria_Incertae_Sedis | Unknown_Family | Acidibacter |  |
| Actinobacteriota | Acidimicrobiia | Microtrichales | Iamiaceae | Iamia | x |
| Acidobacteriota | Vicinamibacteria | Vicinamibacterales | Vicinamibacteraceae | NA |  |
| Myxococcota | Polyangia | Polyangiales | Phaselicystidaceae | Phaselicystis |  |
| Actinobacteriota | Acidimicrobiia | Microtrichales | Ilumatobacteraceae | Ilumatobacter |  |
| Myxococcota | Polyangia | Polyangiales | Polyangiaceae | Aetherobacter |  |
| Actinobacteriota | Actinobacteria | Micrococcales | Cellulomonadaceae | Cellulomonas | x |
| Proteobacteria | Alphaproteobacteria | Azospirillales | NA | NA |  |
| Actinobacteriota | Acidimicrobiia | Microtrichales | Ilumatobacteraceae | NA |  |
| Gemmatimonadota | Gemmatimonadetes | Gemmatimonadales | Gemmatimonadaceae | NA |  |
| Acidobacteriota | Thermoanaerobaculia | Thermoanaerobaculales | Thermoanaerobaculaceae | Subgroup_10 |  |
| Acidobacteriota | Acidobacteriae | Bryobacterales | Bryobacteraceae | Bryobacter |  |
| Proteobacteria | Gammaproteobacteria | Burkholderiales | NA | NA |  |
| Proteobacteria | Alphaproteobacteria | Sphingomonadales | Sphingomonadaceae | NA |  |
| Chloroflexi | OLB14 | NA | NA | NA |  |
| Proteobacteria | Alphaproteobacteria | Rhizobiales | Rhizobiales_Incertae_Sedis | Nordella |  |

Supplementary Table 6. Relative abundance of the ten most abundant protist phyla for each site.

| Site ID | phylum | phylum relative abundance | rank |
| --- | --- | --- | --- |
| 2 | Alveolata | 15.6 | 8 |
| 2 | Chlorophyta | 28.3 | 10 |
| 2 | Discosea | 2.3 | 4 |
| 2 | Evosea | 24.7 | 9 |
| 2 | Opisthokonta | 1.0 | 2 |
| 2 | Prasinodermophyta | 0.8 | 1 |
| 2 | Rhizaria | 14.5 | 7 |
| 2 | Rhodophyta | 1.5 | 3 |
| 2 | Stramenopiles | 5.9 | 6 |
| 2 | Tubulinea | 4.9 | 5 |
| 3 | Alveolata | 23.0 | 10 |
| 3 | Chlorophyta | 10.6 | 7 |
| 3 | Discosea | 3.1 | 3 |
| 3 | Evosea | 22.3 | 9 |
| 3 | Opisthokonta | 0.9 | 1 |
| 3 | Prasinodermophyta | 1.5 | 2 |
| 3 | Rhizaria | 20.6 | 8 |
| 3 | Rhodophyta | 3.3 | 4 |
| 3 | Stramenopiles | 7.3 | 6 |
| 3 | Tubulinea | 6.3 | 5 |
| 4 | Alveolata | 29.5 | 10 |
| 4 | Chlorophyta | 23.8 | 9 |
| 4 | Discosea | 1.0 | 2 |
| 4 | Evosea | 17.1 | 8 |
| 4 | Opisthokonta | 0.9 | 1 |
| 4 | Prasinodermophyta | 1.6 | 3 |
| 4 | Rhizaria | 14.1 | 7 |
| 4 | Rhodophyta | 2.2 | 4 |
| 4 | Stramenopiles | 5.6 | 6 |
| 4 | Tubulinea | 3.8 | 5 |
| 5 | Alveolata | 8.3 | 6 |
| 5 | Chlorophyta | 14.2 | 8 |
| 5 | Discosea | 1.5 | 2 |
| 5 | Evosea | 20.0 | 9 |
| 5 | Opisthokonta | 1.0 | 1 |
| 5 | Prasinodermophyta | 7.1 | 5 |
| 5 | Rhizaria | 20.2 | 10 |
| 5 | Rhodophyta | 4.8 | 3 |
| 5 | Stramenopiles | 11.5 | 7 |
| 5 | Tubulinea | 6.5 | 4 |
| 6 | Alveolata | 18.6 | 9 |
| 6 | Chlorophyta | 14.9 | 7 |
| 6 | Discosea | 2.2 | 4 |
| 6 | Evosea | 18.0 | 8 |
| 6 | Opisthokonta | 0.4 | 1 |
| 6 | Prasinodermophyta | 0.6 | 2 |
| 6 | Rhizaria | 24.1 | 10 |
| 6 | Rhodophyta | 1.1 | 3 |
| 6 | Stramenopiles | 10.3 | 6 |
| 6 | Tubulinea | 8.8 | 5 |
| 7 | Alveolata | 14.5 | 8 |
| 7 | Chlorophyta | 23.3 | 10 |
| 7 | Discosea | 2.6 | 3 |
| 7 | Evosea | 22.9 | 9 |
| 7 | Opisthokonta | 1.4 | 2 |
| 7 | Prasinodermophyta | 1.2 | 1 |
| 7 | Rhizaria | 12.1 | 6 |
| 7 | Rhodophyta | 2.6 | 4 |
| 7 | Stramenopiles | 12.6 | 7 |
| 7 | Tubulinea | 5.9 | 5 |
| 8 | Alveolata | 17.3 | 8 |
| 8 | Chlorophyta | 13.0 | 7 |
| 8 | Discosea | 2.6 | 3 |
| 8 | Evosea | 25.6 | 10 |
| 8 | Opisthokonta | 0.8 | 1 |
| 8 | Prasinodermophyta | 2.9 | 4 |
| 8 | Rhizaria | 20.8 | 9 |
| 8 | Rhodophyta | 2.1 | 2 |
| 8 | Stramenopiles | 7.3 | 6 |
| 8 | Tubulinea | 4.6 | 5 |
| 9 | Alveolata | 26.1 | 10 |
| 9 | Chlorophyta | 19.3 | 9 |
| 9 | Discosea | 2.0 | 4 |
| 9 | Evosea | 13.9 | 7 |
| 9 | Opisthokonta | 0.3 | 1 |
| 9 | Prasinodermophyta | 1.8 | 3 |
| 9 | Rhizaria | 17.2 | 8 |
| 9 | Rhodophyta | 1.2 | 2 |
| 9 | Stramenopiles | 9.9 | 6 |
| 9 | Tubulinea | 7.1 | 5 |
| 10 | Alveolata | 18.6 | 9 |
| 10 | Chlorophyta | 19.6 | 10 |
| 10 | Discosea | 2.1 | 4 |
| 10 | Evosea | 18.2 | 8 |
| 10 | Opisthokonta | 0.8 | 1 |
| 10 | Prasinodermophyta | 1.7 | 2 |
| 10 | Rhizaria | 15.1 | 7 |
| 10 | Rhodophyta | 1.9 | 3 |
| 10 | Stramenopiles | 11.7 | 6 |
| 10 | Tubulinea | 9.9 | 5 |
| 11 | Alveolata | 21.5 | 10 |
| 11 | Chlorophyta | 3.1 | 4 |
| 11 | Discosea | 1.7 | 3 |
| 11 | Evosea | 14.8 | 7 |
| 11 | Opisthokonta | 1.3 | 2 |
| 11 | Prasinodermophyta | 1.3 | 1 |
| 11 | Rhizaria | 17.8 | 8 |
| 11 | Rhodophyta | 5.9 | 5 |
| 11 | Stramenopiles | 18.1 | 9 |
| 11 | Tubulinea | 14.5 | 6 |
| 12 | Alveolata | 41.2 | 10 |
| 12 | Chlorophyta | 5.8 | 6 |
| 12 | Discosea | 1.7 | 3 |
| 12 | Evosea | 16.3 | 8 |
| 12 | Opisthokonta | 0.4 | 1 |
| 12 | Prasinodermophyta | 2.7 | 4 |
| 12 | Rhizaria | 18.6 | 9 |
| 12 | Rhodophyta | 1.4 | 2 |
| 12 | Stramenopiles | 6.5 | 7 |
| 12 | Tubulinea | 4.7 | 5 |
| 13 | Alveolata | 18.5 | 9 |
| 13 | Chlorophyta | 26.9 | 10 |
| 13 | Discosea | 1.7 | 4 |
| 13 | Evosea | 14.2 | 8 |
| 13 | Opisthokonta | 1.6 | 3 |
| 13 | Prasinodermophyta | 0.8 | 1 |
| 13 | Rhizaria | 13.7 | 7 |
| 13 | Rhodophyta | 1.0 | 2 |
| 13 | Stramenopiles | 11.6 | 6 |
| 13 | Tubulinea | 7.5 | 5 |
| 14 | Alveolata | 28.3 | 10 |
| 14 | Chlorophyta | 16.0 | 9 |
| 14 | Discosea | 1.1 | 1 |
| 14 | Evosea | 12.8 | 7 |
| 14 | Opisthokonta | 2.2 | 4 |
| 14 | Prasinodermophyta | 1.5 | 2 |
| 14 | Rhizaria | 15.9 | 8 |
| 14 | Rhodophyta | 1.7 | 3 |
| 14 | Stramenopiles | 12.6 | 6 |
| 14 | Tubulinea | 7.6 | 5 |
| 15 | Alveolata | 61.0 | 10 |
| 15 | Chlorophyta | 2.4 | 5 |
| 15 | Discosea | 1.3 | 3 |
| 15 | Evosea | 8.9 | 8 |
| 15 | Opisthokonta | 0.7 | 1 |
| 15 | Prasinodermophyta | 1.1 | 2 |
| 15 | Rhizaria | 10.8 | 9 |
| 15 | Rhodophyta | 1.5 | 4 |
| 15 | Stramenopiles | 5.9 | 7 |
| 15 | Tubulinea | 5.9 | 6 |
| 16 | Alveolata | 40.6 | 10 |
| 16 | Chlorophyta | 9.3 | 6 |
| 16 | Discosea | 1.9 | 3 |
| 16 | Evosea | 11.0 | 8 |
| 16 | Opisthokonta | 1.9 | 4 |
| 16 | Prasinodermophyta | 1.1 | 1 |
| 16 | Rhizaria | 15.7 | 9 |
| 16 | Rhodophyta | 1.5 | 2 |
| 16 | Stramenopiles | 9.6 | 7 |
| 16 | Tubulinea | 6.8 | 5 |
| 17 | Alveolata | 26.9 | 10 |
| 17 | Chlorophyta | 12.8 | 6 |
| 17 | Discosea | 2.1 | 4 |
| 17 | Evosea | 17.3 | 9 |
| 17 | Opisthokonta | 1.1 | 2 |
| 17 | Prasinodermophyta | 0.9 | 1 |
| 17 | Rhizaria | 14.9 | 8 |
| 17 | Rhodophyta | 1.2 | 3 |
| 17 | Stramenopiles | 13.0 | 7 |
| 17 | Tubulinea | 9.2 | 5 |
| 18 | Alveolata | 25.0 | 10 |
| 18 | Chlorophyta | 13.6 | 7 |
| 18 | Discosea | 1.6 | 2 |
| 18 | Evosea | 16.5 | 8 |
| 18 | Opisthokonta | 1.5 | 1 |
| 18 | Prasinodermophyta | 1.6 | 3 |
| 18 | Rhizaria | 19.4 | 9 |
| 18 | Rhodophyta | 1.7 | 4 |
| 18 | Stramenopiles | 12.8 | 6 |
| 18 | Tubulinea | 5.3 | 5 |
| 19 | Alveolata | 37.1 | 10 |
| 19 | Chlorophyta | 6.3 | 6 |
| 19 | Discosea | 1.8 | 3 |
| 19 | Evosea | 14.8 | 8 |
| 19 | Opisthokonta | 0.9 | 1 |
| 19 | Prasinodermophyta | 1.6 | 2 |
| 19 | Rhizaria | 19.0 | 9 |
| 19 | Rhodophyta | 4.2 | 4 |
| 19 | Stramenopiles | 7.6 | 7 |
| 19 | Tubulinea | 5.9 | 5 |
| 20 | Alveolata | 25.3 | 10 |
| 20 | Chlorophyta | 10.5 | 7 |
| 20 | Discosea | 2.6 | 3 |
| 20 | Evosea | 23.5 | 9 |
| 20 | Opisthokonta | 0.6 | 1 |
| 20 | Prasinodermophyta | 1.3 | 2 |
| 20 | Rhizaria | 14.6 | 8 |
| 20 | Rhodophyta | 3.2 | 4 |
| 20 | Stramenopiles | 8.7 | 6 |
| 20 | Tubulinea | 7.8 | 5 |
| 21 | Alveolata | 22.7 | 10 |
| 21 | Chlorophyta | 13.7 | 7 |
| 21 | Discosea | 1.7 | 4 |
| 21 | Evosea | 18.4 | 9 |
| 21 | Opisthokonta | 0.8 | 1 |
| 21 | Prasinodermophyta | 1.4 | 3 |
| 21 | Rhizaria | 18.2 | 8 |
| 21 | Rhodophyta | 1.2 | 2 |
| 21 | Stramenopiles | 12.0 | 6 |
| 21 | Tubulinea | 7.4 | 5 |
| 22 | Alveolata | 25.5 | 10 |
| 22 | Chlorophyta | 12.0 | 6 |
| 22 | Discosea | 2.7 | 4 |
| 22 | Evosea | 18.2 | 9 |
| 22 | Opisthokonta | 0.4 | 1 |
| 22 | Prasinodermophyta | 1.0 | 2 |
| 22 | Rhizaria | 14.3 | 7 |
| 22 | Rhodophyta | 1.5 | 3 |
| 22 | Stramenopiles | 15.9 | 8 |
| 22 | Tubulinea | 7.7 | 5 |
| 23 | Alveolata | 19.3 | 9 |
| 23 | Chlorophyta | 13.1 | 6 |
| 23 | Discosea | 1.8 | 3 |
| 23 | Evosea | 17.8 | 8 |
| 23 | Opisthokonta | 1.8 | 4 |
| 23 | Prasinodermophyta | 1.7 | 1 |
| 23 | Rhizaria | 19.8 | 10 |
| 23 | Rhodophyta | 1.8 | 2 |
| 23 | Stramenopiles | 13.9 | 7 |
| 23 | Tubulinea | 6.9 | 5 |
| 24 | Alveolata | 26.9 | 10 |
| 24 | Chlorophyta | 16.0 | 8 |
| 24 | Discosea | 2.1 | 4 |
| 24 | Evosea | 13.2 | 7 |
| 24 | Opisthokonta | 0.9 | 1 |
| 24 | Prasinodermophyta | 0.9 | 2 |
| 24 | Rhizaria | 17.5 | 9 |
| 24 | Rhodophyta | 1.4 | 3 |
| 24 | Stramenopiles | 11.5 | 6 |
| 24 | Tubulinea | 7.5 | 5 |
| 25 | Alveolata | 19.8 | 8 |
| 25 | Chlorophyta | 5.7 | 5 |
| 25 | Discosea | 1.9 | 4 |
| 25 | Evosea | 20.6 | 9 |
| 25 | Opisthokonta | 0.8 | 1 |
| 25 | Prasinodermophyta | 1.3 | 3 |
| 25 | Rhizaria | 24.0 | 10 |
| 25 | Rhodophyta | 1.2 | 2 |
| 25 | Stramenopiles | 14.2 | 7 |
| 25 | Tubulinea | 8.8 | 6 |
| 26 | Alveolata | 29.7 | 10 |
| 26 | Chlorophyta | 3.0 | 5 |
| 26 | Discosea | 2.0 | 4 |
| 26 | Evosea | 18.5 | 8 |
| 26 | Opisthokonta | 1.9 | 3 |
| 26 | Prasinodermophyta | 1.5 | 2 |
| 26 | Rhizaria | 20.9 | 9 |
| 26 | Rhodophyta | 1.2 | 1 |
| 26 | Stramenopiles | 12.9 | 7 |
| 26 | Tubulinea | 7.0 | 6 |

Supplementary Table 7. Relative abundance of protist phyla in each site. Colours indicate the four types of phyla combinations: (Group 1: Alveolata, Evosea, and Rhizaria [the first 14 sites]; Group 2: Alveolata, Chlorophyta, and Evosea [the next 5 sites]; Group 3: Alveolata, Chlorophyta, and Rhizaria [the next 3 sites]; Group 4: Chlorophyta, Evosea, Rhizaria [ID5]; group5: Alveolata, Chlorophyta, Evosea [ID11]; Group 6: Alveolata, Evoseas, Stramenopiles [ID22].

| Site ID | Alveolata | Chlorophyta | Evosea | Rhizaria | Stramenopiles | sum |
| --- | --- | --- | --- | --- | --- | --- |
| 2 | 15.6 | 28.3 | 24.7 |  |  | 68.5 |
| 3 | 23.0 |  | 22.3 | 20.6 |  | 65.9 |
| 4 | 29.5 | 23.8 | 17.1 |  |  | 70.3 |
| 5 |  | 14.2 | 20.0 | 20.2 |  | 54.4 |
| 6 | 18.6 |  | 18.0 | 24.1 |  | 60.8 |
| 7 | 14.5 | 23.3 | 22.9 |  |  | 60.7 |
| 8 | 17.3 |  | 25.6 | 20.8 |  | 63.7 |
| 9 | 26.1 | 19.3 |  | 17.2 |  | 62.6 |
| 10 | 18.6 | 19.6 | 18.2 |  |  | 56.4 |
| 11 | 21.5 |  |  | 17.8 | 18.1 | 57.4 |
| 12 | 41.2 |  | 16.3 | 18.6 |  | 76.1 |
| 13 | 18.5 | 26.9 | 14.2 |  |  | 59.7 |
| 14 | 28.3 | 16.0 |  | 15.9 |  | 60.2 |
| 15 | 61.0 |  | 8.9 | 10.8 |  | 80.7 |
| 16 | 40.6 |  | 11.0 | 15.7 |  | 67.3 |
| 17 | 26.9 |  | 17.3 | 14.9 |  | 59.2 |
| 18 | 25.0 |  | 16.5 | 19.4 |  | 61.0 |
| 19 | 37.1 |  | 14.8 | 19.0 |  | 70.9 |
| 20 | 25.3 |  | 23.5 | 14.6 |  | 63.4 |
| 21 | 22.7 |  | 18.4 | 18.2 |  | 59.3 |
| 22 | 25.5 |  | 18.2 |  | 15.9 | 59.5 |
| 23 | 19.3 |  | 17.8 | 19.8 |  | 56.8 |
| 24 | 26.9 | 16.0 |  | 17.5 |  | 60.4 |
| 25 | 19.8 |  | 20.6 | 24.0 |  | 64.4 |
| 26 | 29.7 |  | 18.5 | 20.9 |  | 69.1 |

Supplementary Table 8. Taxonomic assignment of the nine core protist ASVs occurring in all sites.

| supergroup | phylum | class | order | family | genus | species |
| --- | --- | --- | --- | --- | --- | --- |
| Archaeplastida | Chlorophyta | Chlorophyceae | Sphaeropleales | Sphaeropleales | Coelastrella | Coelastrella_striolata |
| TSAR | Stramenopiles | Peronosporomycetes | Peronosporomycetes_X | Peronosporales | Pythium | NA |
| Amoebozoa | Evosea | Variosea | Variosea_X | Filamoebidae | Filamoeba | NA |
| Amoebozoa | Evosea | Variosea | Fractovitellida | Schizoplasmodiidae | Ceratiomyxella | Ceratiomyxella_tahitiensis |
| Archaeplastida | Chlorophyta | Chlorophyceae | Chlamydomonadales | Chlamydomonadales | Chlorosarcinopsis | Chlorosarcinopsis_eremi |
| TSAR | Stramenopiles | Peronosporomycetes | Peronosporomycetes_X | Peronosporales | Pythium | Pythium_parvum |
| Amoebozoa | Evosea | Variosea | Fractovitellida | Schizoplasmodiidae | Nematostelium | Nematostelium_ovatum |
| TSAR | Stramenopiles | Eustigmatophyceae | Eustigmatophyceae_X | Eustigmatophyceae | Vischeria | Vischeria_helvetica |
| TSAR | Rhizaria | Filosa-Sarcomonadea | Cercomonadida | Cercomonadidae | Eocercomonas | NA |

Supplementary Table 9. Total number of unique protist ASVs per site, number of unique ASVs per site and their phylum assignment, and their % with respect to total number of unique ASVs per site.

| site ID | phylum | number of unique ASVs | Total number of unique ASVs | % | rank |
| --- | --- | --- | --- | --- | --- |
| 2 | Alveolata | 37 | 239 | 15.5 | 12 |
| 2 | Breviatea | 1 |  | 0.4 | 1.5 |
| 2 | Chlorophyta | 12 |  | 5.0 | 5.5 |
| 2 | Discoba | 5 |  | 2.1 | 4 |
| 2 | Discosea | 16 |  | 6.7 | 7 |
| 2 | Evosea | 47 |  | 19.7 | 13 |
| 2 | Metamonada | 1 |  | 0.4 | 1.5 |
| 2 | Opisthokonta | 3 |  | 1.3 | 3 |
| 2 | Prasinodermophyta | 12 |  | 5.0 | 5.5 |
| 2 | Rhizaria | 29 |  | 12.1 | 10 |
| 2 | Rhodophyta | 22 |  | 9.2 | 8 |
| 2 | Stramenopiles | 30 |  | 12.6 | 11 |
| 2 | Tubulinea | 24 |  | 10.0 | 9 |
| 3 | Alveolata | 59 | 274 | 21.5 | 15 |
| 3 | Amoebozoa | 1 |  | 0.4 | 1.5 |
| 3 | Apusomonada | 3 |  | 1.1 | 4 |
| 3 | Centroplasthelida | 2 |  | 0.7 | 3 |
| 3 | Chlorophyta | 6 |  | 2.2 | 6.5 |
| 3 | Discoba | 4 |  | 1.5 | 5 |
| 3 | Discosea | 14 |  | 5.1 | 8 |
| 3 | Evosea | 30 |  | 10.9 | 13 |
| 3 | Metamonada | 1 |  | 0.4 | 1.5 |
| 3 | Opisthokonta | 6 |  | 2.2 | 6.5 |
| 3 | Prasinodermophyta | 23 |  | 8.4 | 9 |
| 3 | Rhizaria | 48 |  | 17.5 | 14 |
| 3 | Rhodophyta | 28 |  | 10.2 | 12 |
| 3 | Stramenopiles | 24 |  | 8.8 | 10 |
| 3 | Tubulinea | 25 |  | 9.1 | 11 |
| 4 | Alveolata | 36 | 127 | 28.3 | 16 |
| 4 | Apusomonada | 1 |  | 0.8 | 3.5 |
| 4 | Breviatea | 1 |  | 0.8 | 3.5 |
| 4 | Chlorophyta | 3 |  | 2.4 | 7.5 |
| 4 | Discoba | 1 |  | 0.8 | 3.5 |
| 4 | Discosea | 6 |  | 4.7 | 10.5 |
| 4 | Evosea | 15 |  | 11.8 | 13.5 |
| 4 | Haptophyta | 1 |  | 0.8 | 3.5 |
| 4 | Metamonada | 1 |  | 0.8 | 3.5 |
| 4 | Opisthokonta | 4 |  | 3.1 | 9 |
| 4 | Prasinodermophyta | 6 |  | 4.7 | 10.5 |
| 4 | Rhizaria | 24 |  | 18.9 | 15 |
| 4 | Rhodophyta | 9 |  | 7.1 | 12 |
| 4 | Stramenopiles | 15 |  | 11.8 | 13.5 |
| 4 | Telonemia | 1 |  | 0.8 | 3.5 |
| 4 | Tubulinea | 3 |  | 2.4 | 7.5 |
| 5 | Alveolata | 10 | 271 | 3.7 | 5 |
| 5 | Chlorophyta | 18 |  | 6.6 | 7 |
| 5 | Discoba | 28 |  | 10.3 | 8 |
| 5 | Discosea | 4 |  | 1.5 | 3 |
| 5 | Evosea | 31 |  | 11.4 | 9 |
| 5 | Metamonada | 3 |  | 1.1 | 2 |
| 5 | Opisthokonta | 2 |  | 0.7 | 1 |
| 5 | Prasinodermophyta | 70 |  | 25.8 | 12 |
| 5 | Rhizaria | 32 |  | 11.8 | 10 |
| 5 | Rhodophyta | 53 |  | 19.6 | 11 |
| 5 | Stramenopiles | 14 |  | 5.2 | 6 |
| 5 | Tubulinea | 6 |  | 2.2 | 4 |
| 6 | Alveolata | 28 | 189 | 14.8 | 13 |
| 6 | Amoebozoa | 1 |  | 0.5 | 2 |
| 6 | Breviatea | 1 |  | 0.5 | 2 |
| 6 | Centroplasthelida | 3 |  | 1.6 | 4 |
| 6 | Chlorophyta | 8 |  | 4.2 | 6 |
| 6 | Discoba | 5 |  | 2.6 | 5 |
| 6 | Discosea | 9 |  | 4.8 | 7 |
| 6 | Evosea | 19 |  | 10.1 | 10 |
| 6 | Opisthokonta | 1 |  | 0.5 | 2 |
| 6 | Prasinodermophyta | 20 |  | 10.6 | 11 |
| 6 | Rhizaria | 48 |  | 25.4 | 14 |
| 6 | Rhodophyta | 13 |  | 6.9 | 9 |
| 6 | Stramenopiles | 21 |  | 11.1 | 12 |
| 6 | Tubulinea | 12 |  | 6.3 | 8 |
| 7 | Alveolata | 18 | 141 | 12.8 | 11 |
| 7 | Amoebozoa | 1 |  | 0.7 | 1.5 |
| 7 | Chlorophyta | 6 |  | 4.3 | 5 |
| 7 | Discoba | 4 |  | 2.8 | 4 |
| 7 | Discosea | 9 |  | 6.4 | 6 |
| 7 | Evosea | 22 |  | 15.6 | 12 |
| 7 | Metamonada | 1 |  | 0.7 | 1.5 |
| 7 | Opisthokonta | 3 |  | 2.1 | 3 |
| 7 | Prasinodermophyta | 14 |  | 9.9 | 9 |
| 7 | Rhizaria | 10 |  | 7.1 | 7 |
| 7 | Rhodophyta | 24 |  | 17.0 | 13 |
| 7 | Stramenopiles | 17 |  | 12.1 | 10 |
| 7 | Tubulinea | 12 |  | 8.5 | 8 |
| 8 | Alveolata | 73 | 331 | 22.1 | 15 |
| 8 | Apusomonada | 2 |  | 0.6 | 2.5 |
| 8 | Centroplasthelida | 3 |  | 0.9 | 4 |
| 8 | Chlorophyta | 13 |  | 3.9 | 7 |
| 8 | Discoba | 9 |  | 2.7 | 5.5 |
| 8 | Discosea | 15 |  | 4.5 | 8 |
| 8 | Evosea | 47 |  | 14.2 | 13 |
| 8 | Kathablepharidacea | 1 |  | 0.3 | 1 |
| 8 | Metamonada | 2 |  | 0.6 | 2.5 |
| 8 | Opisthokonta | 9 |  | 2.7 | 5.5 |
| 8 | Prasinodermophyta | 22 |  | 6.6 | 10 |
| 8 | Rhizaria | 51 |  | 15.4 | 14 |
| 8 | Rhodophyta | 30 |  | 9.1 | 11 |
| 8 | Stramenopiles | 36 |  | 10.9 | 12 |
| 8 | Tubulinea | 18 |  | 5.4 | 9 |
| 9 | Alveolata | 27 | 162 | 16.7 | 11 |
| 9 | Breviatea | 1 |  | 0.6 | 1 |
| 9 | Chlorophyta | 3 |  | 1.9 | 2.5 |
| 9 | Discoba | 4 |  | 2.5 | 4 |
| 9 | Discosea | 7 |  | 4.3 | 5 |
| 9 | Evosea | 26 |  | 16.0 | 10 |
| 9 | Opisthokonta | 3 |  | 1.9 | 2.5 |
| 9 | Prasinodermophyta | 16 |  | 9.9 | 8 |
| 9 | Rhizaria | 30 |  | 18.5 | 12 |
| 9 | Rhodophyta | 11 |  | 6.8 | 6 |
| 9 | Stramenopiles | 21 |  | 13.0 | 9 |
| 9 | Tubulinea | 13 |  | 8.0 | 7 |
| 10 | Alveolata | 30 | 218 | 13.8 | 12 |
| 10 | Amoebozoa | 1 |  | 0.5 | 2 |
| 10 | Centroplasthelida | 1 |  | 0.5 | 2 |
| 10 | Chlorophyta | 15 |  | 6.9 | 7.5 |
| 10 | Discoba | 6 |  | 2.8 | 5 |
| 10 | Discosea | 8 |  | 3.7 | 6 |
| 10 | Evosea | 27 |  | 12.4 | 11 |
| 10 | Opisthokonta | 2 |  | 0.9 | 4 |
| 10 | Prasinodermophyta | 21 |  | 9.6 | 9 |
| 10 | Rhizaria | 33 |  | 15.1 | 13 |
| 10 | Rhodelphidia | 1 |  | 0.5 | 2 |
| 10 | Rhodophyta | 34 |  | 15.6 | 14 |
| 10 | Stramenopiles | 24 |  | 11.0 | 10 |
| 10 | Tubulinea | 15 |  | 6.9 | 7.5 |
| 11 | Alveolata | 41 | 240 | 17.1 | 9 |
| 11 | Chlorophyta | 4 |  | 1.7 | 3 |
| 11 | Discoba | 2 |  | 0.8 | 1.5 |
| 11 | Discosea | 8 |  | 3.3 | 5 |
| 11 | Evosea | 34 |  | 14.2 | 7 |
| 11 | Opisthokonta | 2 |  | 0.8 | 1.5 |
| 11 | Prasinodermophyta | 6 |  | 2.5 | 4 |
| 11 | Rhizaria | 39 |  | 16.3 | 8 |
| 11 | Rhodophyta | 17 |  | 7.1 | 6 |
| 11 | Stramenopiles | 42 |  | 17.5 | 10 |
| 11 | Tubulinea | 45 |  | 18.8 | 11 |
| 12 | Alveolata | 43 | 213 | 20.2 | 13 |
| 12 | Apusomonada | 2 |  | 0.9 | 3.5 |
| 12 | Centroplasthelida | 1 |  | 0.5 | 1 |
| 12 | Chlorophyta | 2 |  | 0.9 | 3.5 |
| 12 | Discoba | 2 |  | 0.9 | 3.5 |
| 12 | Discosea | 14 |  | 6.6 | 7 |
| 12 | Evosea | 27 |  | 12.7 | 10 |
| 12 | Opisthokonta | 2 |  | 0.9 | 3.5 |
| 12 | Prasinodermophyta | 9 |  | 4.2 | 6 |
| 12 | Rhizaria | 42 |  | 19.7 | 12 |
| 12 | Rhodophyta | 20 |  | 9.4 | 9 |
| 12 | Stramenopiles | 34 |  | 16.0 | 11 |
| 12 | Tubulinea | 15 |  | 7.0 | 8 |
| 13 | Alveolata | 32 | 210 | 15.2 | 14 |
| 13 | Amoebozoa | 1 |  | 0.5 | 2.5 |
| 13 | Centroplasthelida | 1 |  | 0.5 | 2.5 |
| 13 | Chlorophyta | 14 |  | 6.7 | 9 |
| 13 | Discoba | 4 |  | 1.9 | 6 |
| 13 | Discosea | 6 |  | 2.9 | 7 |
| 13 | Evosea | 20 |  | 9.5 | 10.5 |
| 13 | Metamonada | 2 |  | 1.0 | 5 |
| 13 | Opisthokonta | 1 |  | 0.5 | 2.5 |
| 13 | Prasinodermophyta | 20 |  | 9.5 | 10.5 |
| 13 | Rhizaria | 44 |  | 21.0 | 15 |
| 13 | Rhodophyta | 13 |  | 6.2 | 8 |
| 13 | Stramenopiles | 30 |  | 14.3 | 13 |
| 13 | Streptophyta | 1 |  | 0.5 | 2.5 |
| 13 | Tubulinea | 21 |  | 10.0 | 12 |
| 14 | Alveolata | 34 | 158 | 21.5 | 12 |
| 14 | Centroplasthelida | 1 |  | 0.6 | 2 |
| 14 | Chlorophyta | 4 |  | 2.5 | 4 |
| 14 | Discoba | 1 |  | 0.6 | 2 |
| 14 | Discosea | 7 |  | 4.4 | 6 |
| 14 | Evosea | 15 |  | 9.5 | 8 |
| 14 | Opisthokonta | 1 |  | 0.6 | 2 |
| 14 | Prasinodermophyta | 6 |  | 3.8 | 5 |
| 14 | Rhizaria | 30 |  | 19.0 | 11 |
| 14 | Rhodophyta | 12 |  | 7.6 | 7 |
| 14 | Stramenopiles | 27 |  | 17.1 | 10 |
| 14 | Tubulinea | 20 |  | 12.7 | 9 |
| 15 | Alveolata | 37 | 201 | 18.4 | 11 |
| 15 | Amoebozoa | 1 |  | 0.5 | 1 |
| 15 | Chlorophyta | 5 |  | 2.5 | 3.5 |
| 15 | Discoba | 3 |  | 1.5 | 2 |
| 15 | Discosea | 13 |  | 6.5 | 5 |
| 15 | Evosea | 18 |  | 9.0 | 8 |
| 15 | Opisthokonta | 5 |  | 2.5 | 3.5 |
| 15 | Prasinodermophyta | 14 |  | 7.0 | 6 |
| 15 | Rhizaria | 39 |  | 19.4 | 12 |
| 15 | Rhodophyta | 15 |  | 7.5 | 7 |
| 15 | Stramenopiles | 31 |  | 15.4 | 10 |
| 15 | Tubulinea | 20 |  | 10.0 | 9 |
| 16 | Alveolata | 37 | 180 | 20.6 | 13 |
| 16 | Apusomonada | 1 |  | 0.6 | 1 |
| 16 | Centroplasthelida | 2 |  | 1.1 | 2.5 |
| 16 | Chlorophyta | 4 |  | 2.2 | 4.5 |
| 16 | Discoba | 4 |  | 2.2 | 4.5 |
| 16 | Discosea | 12 |  | 6.7 | 7 |
| 16 | Evosea | 21 |  | 11.7 | 10 |
| 16 | Opisthokonta | 2 |  | 1.1 | 2.5 |
| 16 | Prasinodermophyta | 14 |  | 7.8 | 8 |
| 16 | Rhizaria | 30 |  | 16.7 | 12 |
| 16 | Rhodophyta | 19 |  | 10.6 | 9 |
| 16 | Stramenopiles | 26 |  | 14.4 | 11 |
| 16 | Tubulinea | 7 |  | 3.9 | 6 |
| 17 | Alveolata | 15 | 145 | 10.3 | 12 |
| 17 | Amoebozoa | 1 |  | 0.7 | 3 |
| 17 | Apusomonada | 2 |  | 1.4 | 6 |
| 17 | Breviatea | 1 |  | 0.7 | 3 |
| 17 | Chlorophyta | 3 |  | 2.1 | 7 |
| 17 | Discosea | 9 |  | 6.2 | 9 |
| 17 | Evosea | 16 |  | 11.0 | 13 |
| 17 | Haptophyta | 1 |  | 0.7 | 3 |
| 17 | Opisthokonta | 5 |  | 3.4 | 8 |
| 17 | Prasinodermophyta | 14 |  | 9.7 | 11 |
| 17 | Rhizaria | 23 |  | 15.9 | 16 |
| 17 | Rhodophyta | 18 |  | 12.4 | 14 |
| 17 | Rigifilida | 1 |  | 0.7 | 3 |
| 17 | Stramenopiles | 22 |  | 15.2 | 15 |
| 17 | Streptophyta | 1 |  | 0.7 | 3 |
| 17 | Tubulinea | 13 |  | 9.0 | 10 |
| 18 | Alveolata | 39 | 215 | 18.1 | 12 |
| 18 | Amoebozoa | 3 |  | 1.4 | 2 |
| 18 | Centroplasthelida | 1 |  | 0.5 | 1 |
| 18 | Chlorophyta | 7 |  | 3.3 | 5.5 |
| 18 | Discoba | 4 |  | 1.9 | 3 |
| 18 | Discosea | 10 |  | 4.7 | 7 |
| 18 | Evosea | 25 |  | 11.6 | 10 |
| 18 | Opisthokonta | 5 |  | 2.3 | 4 |
| 18 | Prasinodermophyta | 7 |  | 3.3 | 5.5 |
| 18 | Rhizaria | 35 |  | 16.3 | 11 |
| 18 | Rhodophyta | 16 |  | 7.4 | 8 |
| 18 | Stramenopiles | 42 |  | 19.5 | 13 |
| 18 | Tubulinea | 21 |  | 9.8 | 9 |
| 19 | Alveolata | 20 | 139 | 14.4 | 11 |
| 19 | Amoebozoa | 1 |  | 0.7 | 1.5 |
| 19 | Centroplasthelida | 2 |  | 1.4 | 3 |
| 19 | Chlorophyta | 5 |  | 3.6 | 4 |
| 19 | Discosea | 8 |  | 5.8 | 6.5 |
| 19 | Evosea | 19 |  | 13.7 | 10 |
| 19 | Metamonada | 1 |  | 0.7 | 1.5 |
| 19 | Opisthokonta | 6 |  | 4.3 | 5 |
| 19 | Prasinodermophyta | 8 |  | 5.8 | 6.5 |
| 19 | Rhizaria | 21 |  | 15.1 | 12.5 |
| 19 | Rhodophyta | 15 |  | 10.8 | 9 |
| 19 | Stramenopiles | 21 |  | 15.1 | 12.5 |
| 19 | Tubulinea | 12 |  | 8.6 | 8 |
| 20 | Alveolata | 43 | 313 | 13.7 | 13 |
| 20 | Amoebozoa | 3 |  | 1.0 | 3.5 |
| 20 | Apusomonada | 3 |  | 1.0 | 3.5 |
| 20 | Chlorophyta | 14 |  | 4.5 | 7.5 |
| 20 | Discoba | 14 |  | 4.5 | 7.5 |
| 20 | Discosea | 10 |  | 3.2 | 6 |
| 20 | Evosea | 44 |  | 14.1 | 14 |
| 20 | Haptophyta | 1 |  | 0.3 | 1.5 |
| 20 | Opisthokonta | 6 |  | 1.9 | 5 |
| 20 | Prasinodermophyta | 29 |  | 9.3 | 10.5 |
| 20 | Rhizaria | 54 |  | 17.3 | 15 |
| 20 | Rhodophyta | 36 |  | 11.5 | 12 |
| 20 | Stramenopiles | 29 |  | 9.3 | 10.5 |
| 20 | Telonemia | 1 |  | 0.3 | 1.5 |
| 20 | Tubulinea | 26 |  | 8.3 | 9 |
| 21 | Alveolata | 26 | 148 | 17.6 | 13.5 |
| 21 | Amoebozoa | 1 |  | 0.7 | 2.5 |
| 21 | Chlorophyta | 2 |  | 1.4 | 5.5 |
| 21 | Discoba | 1 |  | 0.7 | 2.5 |
| 21 | Discosea | 5 |  | 3.4 | 7 |
| 21 | Evosea | 15 |  | 10.1 | 9.5 |
| 21 | Opisthokonta | 2 |  | 1.4 | 5.5 |
| 21 | Prasinodermophyta | 10 |  | 6.8 | 8 |
| 21 | Rhizaria | 26 |  | 17.6 | 13.5 |
| 21 | Rhodophyta | 15 |  | 10.1 | 9.5 |
| 21 | Stramenopiles | 22 |  | 14.9 | 12 |
| 21 | Streptophyta | 1 |  | 0.7 | 2.5 |
| 21 | Telonemia | 1 |  | 0.7 | 2.5 |
| 21 | Tubulinea | 21 |  | 14.2 | 11 |
| 22 | Alveolata | 33 | 206 | 16.0 | 13 |
| 22 | Apusomonada | 1 |  | 0.5 | 1.5 |
| 22 | Centroplasthelida | 2 |  | 1.0 | 3.5 |
| 22 | Chlorophyta | 7 |  | 3.4 | 7 |
| 22 | Discoba | 1 |  | 0.5 | 1.5 |
| 22 | Discosea | 20 |  | 9.7 | 9 |
| 22 | Evosea | 29 |  | 14.1 | 12 |
| 22 | Metamonada | 2 |  | 1.0 | 3.5 |
| 22 | Opisthokonta | 4 |  | 1.9 | 5 |
| 22 | Prasinodermophyta | 5 |  | 2.4 | 6 |
| 22 | Rhizaria | 26 |  | 12.6 | 11 |
| 22 | Rhodophyta | 8 |  | 3.9 | 8 |
| 22 | Stramenopiles | 46 |  | 22.3 | 14 |
| 22 | Tubulinea | 22 |  | 10.7 | 10 |
| 23 | Alveolata | 36 | 355 | 10.1 | 10 |
| 23 | Apusomonada | 2 |  | 0.6 | 1 |
| 23 | Centroplasthelida | 6 |  | 1.7 | 3 |
| 23 | Chlorophyta | 14 |  | 3.9 | 6 |
| 23 | Discoba | 9 |  | 2.5 | 4 |
| 23 | Discosea | 10 |  | 2.8 | 5 |
| 23 | Evosea | 59 |  | 16.6 | 12 |
| 23 | Opisthokonta | 5 |  | 1.4 | 2 |
| 23 | Prasinodermophyta | 20 |  | 5.6 | 7 |
| 23 | Rhizaria | 81 |  | 22.8 | 13 |
| 23 | Rhodophyta | 33 |  | 9.3 | 9 |
| 23 | Stramenopiles | 50 |  | 14.1 | 11 |
| 23 | Tubulinea | 30 |  | 8.5 | 8 |
| 24 | Alveolata | 44 | 309 | 14.2 | 14 |
| 24 | Amoebozoa | 1 |  | 0.3 | 2 |
| 24 | Apusomonada | 2 |  | 0.6 | 4.5 |
| 24 | Breviatea | 1 |  | 0.3 | 2 |
| 24 | Chlorophyta | 18 |  | 5.8 | 8.5 |
| 24 | Discoba | 9 |  | 2.9 | 7 |
| 24 | Discosea | 18 |  | 5.8 | 8.5 |
| 24 | Evosea | 27 |  | 8.7 | 12 |
| 24 | Haptophyta | 1 |  | 0.3 | 2 |
| 24 | Metamonada | 3 |  | 1.0 | 6 |
| 24 | Opisthokonta | 2 |  | 0.6 | 4.5 |
| 24 | Prasinodermophyta | 19 |  | 6.1 | 10 |
| 24 | Rhizaria | 60 |  | 19.4 | 16 |
| 24 | Rhodophyta | 34 |  | 11.0 | 13 |
| 24 | Stramenopiles | 45 |  | 14.6 | 15 |
| 24 | Tubulinea | 25 |  | 8.1 | 11 |
| 25 | Alveolata | 52 | 330 | 15.8 | 12 |
| 25 | Amoebozoa | 1 |  | 0.3 | 1 |
| 25 | Centroplasthelida | 2 |  | 0.6 | 2.5 |
| 25 | Chlorophyta | 15 |  | 4.5 | 7 |
| 25 | Discoba | 4 |  | 1.2 | 4 |
| 25 | Discosea | 15 |  | 4.5 | 7 |
| 25 | Evosea | 36 |  | 10.9 | 10 |
| 25 | Metamonada | 2 |  | 0.6 | 2.5 |
| 25 | Opisthokonta | 8 |  | 2.4 | 5 |
| 25 | Prasinodermophyta | 15 |  | 4.5 | 7 |
| 25 | Rhizaria | 53 |  | 16.1 | 13 |
| 25 | Rhodophyta | 27 |  | 8.2 | 9 |
| 25 | Stramenopiles | 62 |  | 18.8 | 14 |
| 25 | Tubulinea | 38 |  | 11.5 | 11 |
| 26 | Alveolata | 57 | 257 | 22.2 | 15 |
| 26 | Amoebozoa | 1 |  | 0.4 | 1.5 |
| 26 | Apusomonada | 1 |  | 0.4 | 1.5 |
| 26 | Centroplasthelida | 3 |  | 1.2 | 4 |
| 26 | Chlorophyta | 8 |  | 3.1 | 6 |
| 26 | Discoba | 8 |  | 3.1 | 6 |
| 26 | Discosea | 15 |  | 5.8 | 10 |
| 26 | Evosea | 27 |  | 10.5 | 12 |
| 26 | Metamonada | 2 |  | 0.8 | 3 |
| 26 | Opisthokonta | 8 |  | 3.1 | 6 |
| 26 | Prasinodermophyta | 12 |  | 4.7 | 8.5 |
| 26 | Rhizaria | 40 |  | 15.6 | 13 |
| 26 | Rhodophyta | 12 |  | 4.7 | 8.5 |
| 26 | Stramenopiles | 42 |  | 16.3 | 14 |
| 26 | Tubulinea | 21 |  | 8.2 | 11 |

Supplementary Table 10. Relative abundance of fungal phyla in each site

| site ID | Ascomycota | Basidiomycota | Mortierellomycota | Rozellomycota | total relative abundance |
| --- | --- | --- | --- | --- | --- |
| 1 | 81.4 | 11.0 |  | 3.6 | 96.1 |
| 3 | 79.2 | 5.6 | 6.3 |  | 91.0 |
| 4 | 89.6 | 6.8 |  | 1.5 | 97.8 |
| 5 | 78.6 | 9.2 | 4.8 |  | 92.6 |
| 6 | 65.1 | 11.0 | 13.6 |  | 89.7 |
| 7 | 88.6 | 3.6 | 5.3 |  | 97.4 |
| 8 | 70.1 | 23.2 | 4.4 |  | 97.6 |
| 11 | 80.9 | 8.8 | 6.9 |  | 96.6 |
| 12 | 76.7 | 11.8 | 7.7 |  | 96.2 |
| 13 | 77.8 | 10.1 | 6.1 |  | 94.0 |
| 14 | 85.4 | 7.1 | 4.2 |  | 96.7 |
| 16 | 65.8 | 10.4 | 16.3 |  | 92.5 |
| 17 | 66.9 | 19.0 | 5.8 |  | 91.6 |
| 18 | 69.9 | 11.0 | 10.7 |  | 91.6 |
| 21 | 72.8 | 8.4 | 11.9 |  | 93.1 |
| 22 | 73.9 | 5.3 | 12.5 |  | 91.7 |
| 24 | 64.2 | 13.2 | 13.5 |  | 90.9 |
| 25 | 77.4 | 8.1 | 8.6 |  | 94.1 |
| 26 | 84.1 | 6.1 | 6.1 |  | 96.4 |

Supplementary Table 11. Taxonomic assignment of the 13 core fungal ASVs occurring in all sites.

| Phylum | Class | Order | Family | Genus | Species |
| --- | --- | --- | --- | --- | --- |
| p__Ascomycota | c__Sordariomycetes | o__Xylariales | f__Bartaliniaceae | g__Truncatella | s__angustata |
| p__Ascomycota | c__Sordariomycetes | o__Glomerellales | f__Plectosphaerellaceae | g__Verticillium | s__dahliae |
| p__Mortierellomycota | c__Mortierellomycetes | o__Mortierellales | f__Mortierellaceae | g__Podila | s__epicladia |
| p__Ascomycota | c__Sordariomycetes | o__Hypocreales | f__Nectriaceae | g__Fusarium | s__tricinctum |
| p__Ascomycota | c__Sordariomycetes | o__Hypocreales | f__Nectriaceae | g__Fusarium | s__equiseti |
| p__Ascomycota | c__Dothideomycetes | o__Capnodiales | f__Cladosporiaceae | g__Cladosporium | s__basi-inflatum |
| p__Basidiomycota | c__Tremellomycetes | o__Filobasidiales | f__Piskurozymaceae | g__Solicoccozyma | s__aeria |
| p__Ascomycota | c__Dothideomycetes | o__Pleosporales | f__Pleosporaceae | g__Alternaria | NA |
| p__Ascomycota | c__Sordariomycetes | o__Hypocreales | f__Nectriaceae | g__Neocosmospora | s__rubicola |
| p__Ascomycota | c__Leotiomycetes | o__Helotiales | f__Helotiaceae | g__Tetracladium | s__marchalianum |
| p__Ascomycota | c__Sordariomycetes | o__Hypocreales | f__Nectriaceae | g__Thelonectria | NA |
| p__Ascomycota | c__Sordariomycetes | o__Hypocreales | f__Nectriaceae | g__Dactylonectria | s__macrodidyma |
| p__Ascomycota | c__Eurotiomycetes | o__Chaetothyriales | f__Herpotrichiellaceae | g__Exophiala | s__radicis |

Supplementary Table 12. Total number of unique fungal ASVs per site, number of unique ASVs per site and their phylum assignment, and their % with respect to total number of unique ASVs per site.

| Site ID | phylum | number of unique ASVs per phylum | total number of unique ASVs | % | rank |
| --- | --- | --- | --- | --- | --- |
| 1 | Ascomycota | 2 | 4 | 50.0 | 1.5 |
| 1 | Basidiomycota | 2 |  | 50.0 | 1.5 |
| 3 | Ascomycota | 4 | 8 | 50.0 | 3 |
| 3 | Basidiomycota | 3 |  | 37.5 | 2 |
| 3 | Glomeromycota | 1 |  | 12.5 | 1 |
| 4 | Ascomycota | 6 | 10 | 60.0 | 3 |
| 4 | Basidiomycota | 3 |  | 30.0 | 2 |
| 4 | Chytridiomycota | 1 |  | 10.0 | 1 |
| 5 | Ascomycota | 3 | 3 | 100.0 | 1 |
| 6 | Ascomycota | 5 | 9 | 55.6 | 3 |
| 6 | Basidiomycota | 3 |  | 33.3 | 2 |
| 6 | Chytridiomycota | 1 |  | 11.1 | 1 |
| 7 | Ascomycota | 14 | 17 | 82.4 | 3 |
| 7 | Basidiomycota | 2 |  | 11.8 | 2 |
| 7 | Entorrhizomycota | 1 |  | 5.9 | 1 |
| 8 | Ascomycota | 13 | 20 | 65.0 | 4 |
| 8 | Basidiomycota | 5 |  | 25.0 | 3 |
| 8 | Blastocladiomycota | 1 |  | 5.0 | 1.5 |
| 8 | Chytridiomycota | 1 |  | 5.0 | 1.5 |
| 11 | Ascomycota | 8 | 11 | 72.7 | 4 |
| 11 | Basidiomycota | 1 |  | 9.1 | 2 |
| 11 | Chytridiomycota | 1 |  | 9.1 | 2 |
| 11 | Mortierellomycota | 1 |  | 9.1 | 2 |
| 12 | Ascomycota | 7 | 17 | 41.2 | 1 |
| 12 | Basidiomycota | 10 |  | 58.8 | 2 |
| 13 | Ascomycota | 4 | 8 | 50.0 | 3 |
| 13 | Basidiomycota | 3 |  | 37.5 | 2 |
| 13 | Chytridiomycota | 1 |  | 12.5 | 1 |
| 14 | Ascomycota | 3 | 6 | 50.0 | 3 |
| 14 | Basidiomycota | 2 |  | 33.3 | 2 |
| 14 | Mucoromycota | 1 |  | 16.7 | 1 |
| 16 | Ascomycota | 2 |  | 33.3 | 2 |
| 16 | Basidiomycota | 3 |  | 50.0 | 3 |
| 16 | Kickxellomycota | 1 |  | 16.7 | 1 |
| 17 | Ascomycota | 6 | 9 | 66.7 | 2 |
| 17 | Basidiomycota | 3 |  | 33.3 | 1 |
| 18 | Ascomycota | 10 | 17 | 58.8 | 3 |
| 18 | Basidiomycota | 6 |  | 35.3 | 2 |
| 18 | Mortierellomycota | 1 |  | 5.9 | 1 |
| 21 | Ascomycota | 3 | 9 | 33.3 | 4.5 |
| 21 | Basidiomycota | 3 |  | 33.3 | 4.5 |
| 21 | Chytridiomycota | 1 |  | 11.1 | 2 |
| 21 | Neocallimastigomycota | 1 |  | 11.1 | 2 |
| 21 | Olpidiomycota | 1 |  | 11.1 | 2 |
| 22 | Ascomycota | 3 | 5 | 60.0 | 2 |
| 22 | Basidiomycota | 2 |  | 40.0 | 1 |
| 24 | Ascomycota | 6 | 12 | 50.0 | 3 |
| 24 | Basidiomycota | 5 |  | 41.7 | 2 |
| 24 | Rozellomycota | 1 |  | 8.3 | 1 |
| 25 | Ascomycota | 11 | 18 | 61.1 | 4 |
| 25 | Basidiomycota | 5 |  | 27.8 | 3 |
| 25 | Chytridiomycota | 1 |  | 5.6 | 1.5 |
| 25 | Zoopagomycota | 1 |  | 5.6 | 1.5 |
| 26 | Ascomycota | 4 | 10 | 40.0 | 6 |
| 26 | Basidiomycota | 1 |  | 10.0 | 2.5 |
| 26 | Chytridiomycota | 1 |  | 10.0 | 2.5 |
| 26 | Mortierellomycota | 2 |  | 20.0 | 5 |
| 26 | Olpidiomycota | 1 |  | 10.0 | 2.5 |
| 26 | Rozellomycota | 1 |  | 10.0 | 2.5 |

Supplementary Table 13. % contribution of turnover and balanced variation in species abundance, respectively, to beta-diversity of the four organismal groups.

|  | prokayotes | protists | fungi |
| --- | --- | --- | --- |
| % turnover based on presence/absence | 92.7 % | 97.7 % | 95 % |
| % balanced variation in species abundance based on abundance | 100 % | 100 % | 100 % |
